# Neuronal SNAP-23 is critical for synaptic plasticity and spatial memory independently of NMDA receptor regulation

**DOI:** 10.1016/j.isci.2023.106664

**Published:** 2023-04-13

**Authors:** Mengjia Huang, Na-Ryum Bin, Jayant Rai, Ke Ma, Chun Hin Chow, Sarah Eide, Hidekiyo Harada, Jianbing Xiao, Daorong Feng, Hong-Shuo Sun, Zhong-Ping Feng, Herbert Y. Gaisano, Jeffrey E. Pessin, Philippe P. Monnier, Kenichi Okamoto, Liang Zhang, Shuzo Sugita

**Affiliations:** 1 Division of Experimental & Translational Neuroscience, Krembil Brain Institute, University Health Network, Toronto, ON M5T 0S8, Canada; 2 Department of Physiology, Temerty Faculty of Medicine, University of Toronto, Toronto, ON M5S 1A8, Canada; 3 Lunenfeld-Tanenbaum Research Institute, Sinai Health System, Toronto, ON M5G1X5, Canada; 4 Department of Molecular Genetics, Temerty Faculty of Medicine, University of Toronto, Toronto, ON M5S 1A8, Canada; 5 Department of Pediatrics, The First Hospital of Jilin University, Changchun 130021, China; 6 Donald K. Johnson Eye Institute, University Health Network, Toronto, ON M5T 0S8, Canada; 7 Department of Anatomy, Harbin Medical University, Harbin 150081, China; 8 Department of Medicine, Albert Einstein College of Medicine, Bronx, NY 10461, USA; 9 Department of Anatomy, Temerty Faculty of Medicine, University of Toronto, Toronto, ON M5S 1A8, Canada; 10 Toronto General Hospital Research Institute, University Health Network, Toronto, ON M5G 2C4, Canada; 11 Department of Medicine, Temerty Faculty of Medicine, University of Toronto, Toronto, ON M5S 1A8, Canada; 12 Department of Molecular Pharmacology, Albert Einstein College of Medicine, Bronx, NY 10461, USA; 13 Department of Ophthalmology & Vision Sciences, University of Toronto, Toronto, ON M5S 1A8, Canada

**Keywords:** Molecular biology, Neuroscience, Molecular neuroscience

## Abstract

SNARE-mediated membrane fusion plays a crucial role in presynaptic vesicle exocytosis and also in postsynaptic receptor delivery. The latter is considered particularly important for synaptic plasticity and learning and memory, yet the identity of the key SNARE proteins remains elusive. Here, we investigate the role of neuronal synaptosomal-associated protein-23 (SNAP-23) by analyzing pyramidal-neuron specific SNAP-23 conditional knockout (cKO) mice. Electrophysiological analysis of SNAP-23 deficient neurons using acute hippocampal slices showed normal basal neurotransmission in CA3-CA1 synapses with unchanged AMPA and NMDA currents. Nevertheless, we found theta-burst stimulation-induced long-term potentiation (LTP) was vastly diminished in SNAP-23 cKO slices. Moreover, unlike syntaxin-4 cKO mice where both basal neurotransmission and LTP decrease manifested changes in a broad set of behavioral tasks, deficits of SNAP-23 cKO are more limited to spatial memory. Our data reveal that neuronal SNAP-23 is selectively crucial for synaptic plasticity and spatial memory without affecting basal glutamate receptor function.

## Highlights

• Pyramidal neuron specific knockout (cKO) of SNAP-23 impairs hippocampal LTP
• Basal synaptic transmission and NMDAR currents are unaltered in SNAP-23 cKO mice
• SNAP-23 cKO leads to impaired spatial learning and memory
• Syntaxin-4 cKO leads to broad behavioral deficits

## Graphical abstract

**Figure:**
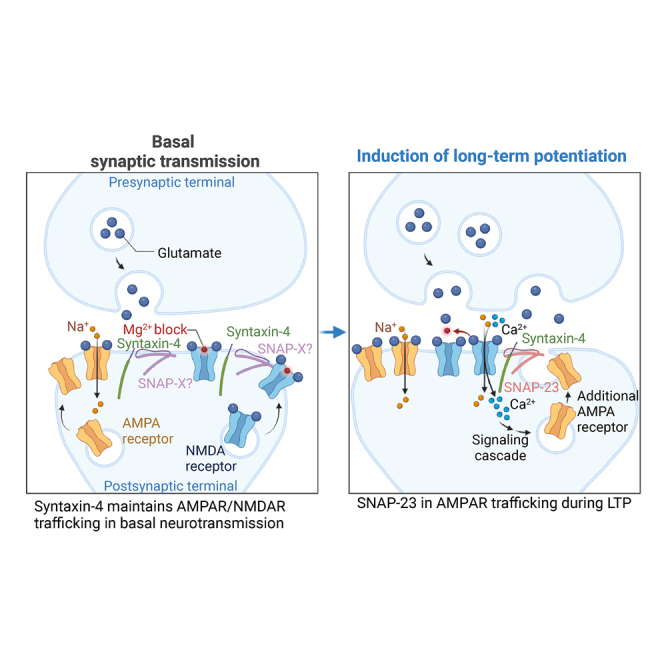

## Introduction

Neuronal communication is essential for the function of the brain. Synaptic communications between neurons are dependent on a cellular process called exocytosis, wherein cargo—such as neurotransmitters stored in presynaptic vesicles—is delivered to the extracellular environment. Glutamate is the main excitatory neurotransmitter in the mammalian central nervous system. Presynaptically released glutamate binds to postsynaptic ionotropic or metabotropic receptors, which then exert their downstream effects to elicit activation of postsynaptic neurons.

Presynaptic vesicle exocytosis depends on the soluble NSF-attachment protein receptor (SNARE) complex, a ternary protein complex, in which vesicular R-SNARE synaptobrevin/vesicle-associated membrane protein (VAMP) binds to target Q-SNAREs syntaxin-1 and SNAP-25 into a complex that drives membrane fusion resulting in rapid neurotransmitter releases.^1^^,^^2^^,^^3^ Recent evidence suggests that vesicular exocytosis is also involved in the regulation of postsynaptic glutamate receptors, α-amino-3-hydroxy-5-methyl-4-isoxazolepropionic acid (AMPA) receptors, and *N*-Methyl-D-aspartic acid (NMDA) receptors.^4^ These receptors are known to undergo rounds of receptor recycling, being inserted or removed from the postsynaptic membrane, thereby controlling synaptic strength and plasticity as required to maintain proper neural communications and functions.^5^^,^^6^^,^^7^^,^^8^^,^^9^^,^^10^^,^^11^^,^^12^

Long-term potentiation (LTP) is the most widely studied form of synaptic plasticity and is believed to be the local circuitry correlate of learning and memory. LTP induction by high-frequency or theta-burst stimulation is known to change postsynaptic receptor numbers and composition.^13^ LTP results in NMDA receptor (NMDAR) activation, which further triggers AMPA receptor (AMPAR) insertion into the membrane of postsynaptic neurons via exocytosis, which is thought to be dependent on the SNARE complex. Moreover, there is evidence suggesting that the exocytosis of ionotropic glutamate receptors to the postsynaptic side is critical for the maintenance phase of LTP.^14^^,^^15^^,^^16^ Similarly, the endocytosis of ionotropic glutamate receptors on the postsynaptic side is critical for long-term depression (LTD).^17^^,^^18^ In addition to controlling the number of surface postsynaptic receptors, changes to the stoichiometry of postsynaptic neurotransmitter receptors have also been observed.^18^ Moreover, it has been shown that not only do AMPA receptor numbers change during events of synaptic plasticity^14^^,^^15^^,^^16^^,^^17^ but NMDA receptor subunit changes can also occur, with evidence suggesting that the delivery rate varies among the receptor subunits.^15^^,^^19^

SNARE proteins are hypothesized to be involved in receptor delivery by mediating vesicle fusion and trafficking at the postsynaptic membrane.^14^^,^^19^^,^^20^ Nonetheless, the precise identities of SNARE proteins implicated in this process remain unclear. The synaptosomal-associated protein (SNAP) protein is a t-SNARE protein involved in vesicle fusion. There are 4 SNAP isoforms, SNAP-23, SNAP-25, SNAP-29, and SNAP-47, with nomenclature based on their molecular weight. SNAP-25 is the neuronal isoform^21^ while SNAP-23, 29, and 47 are ubiquitous isoforms.^22^^,^^23^ The lethality of a global SNAP-23 knockout and other isoforms in mice has led to approaches such as using shRNA-mediated acute knockdown or heterozygous mice to be employed for functional analyses. As such, the role of SNAP-23 in the regulation of postsynaptic AMPA and NMDA currents has been particularly controversial. For example, some argue for a role of SNAP-23 in NMDAR trafficking,^24^ while others counter that SNAP-25 plays this role.^25^ More importantly, the lack of an *in vivo* animal model precludes any investigation of the behavioral consequences of SNAP-23 deletion.

In this study, we use CaMKIIα-Cre to generate a pyramidal neuron-specific conditional SNAP-23 KO (cKO) mouse.^26^^,^^27^^,^^28^^,^^29^ Using this cKO mouse line, we analyze the potential postsynaptic function of SNAP-23 *in vitro* and *in vivo* using a combination of histological, electrophysiological, and behavioral approaches. We show that SNAP-23 cKO results in defects of synaptic plasticity without impairing basal transmission. We also show the correlation between synaptic deficits and behavioral changes by comparing the phenotypes of SNAP-23 cKO mice with that of syntaxin-4 cKO mice, which have reduced basal transmission as well as LTP.^28^ Our results demonstrate that the behavioral changes of SNAP-23 cKO are more selective than that of syntaxin-4 cKO.

## Results

### Generation of pyramidal neuron-specific SNAP-23 cKO mice

SNAP-23 null and germline SNAP-23 deletion mice result in pre-implantation embryonic lethality prior to embryonic day 3.5.^27^^,^^30^ Therefore, we took advantage of a conditional knockout (cKO) system to circumvent the embryonic lethality of global knockout models in order to test the role of SNAP-23. To do this, a pyramidal neuron-specific knockout (KO) of SNAP-23 was generated by crossing SNAP-23 flox/flox mice (SNAP23^tm1Jpes^), in which exons 3 and 4 were flanked by *loxP* sites with a CaMKIIα-Cre mouse line. CaMKIIα-Cre is strongly expressed in pyramidal neurons, particularly in the CA1 region, of the hippocampus.^26^^,^^31^ This pyramidal neuron-specific SNAP-23 conditional KO (SNAP-23 cKO; full name SNAP23^tm1Jpes^; B6.Cg-Tg(Camk2a-cre)T29-1Stl/J) line appeared visually normal at weaning and the mice were viable and fertile.

**Figure 1:**
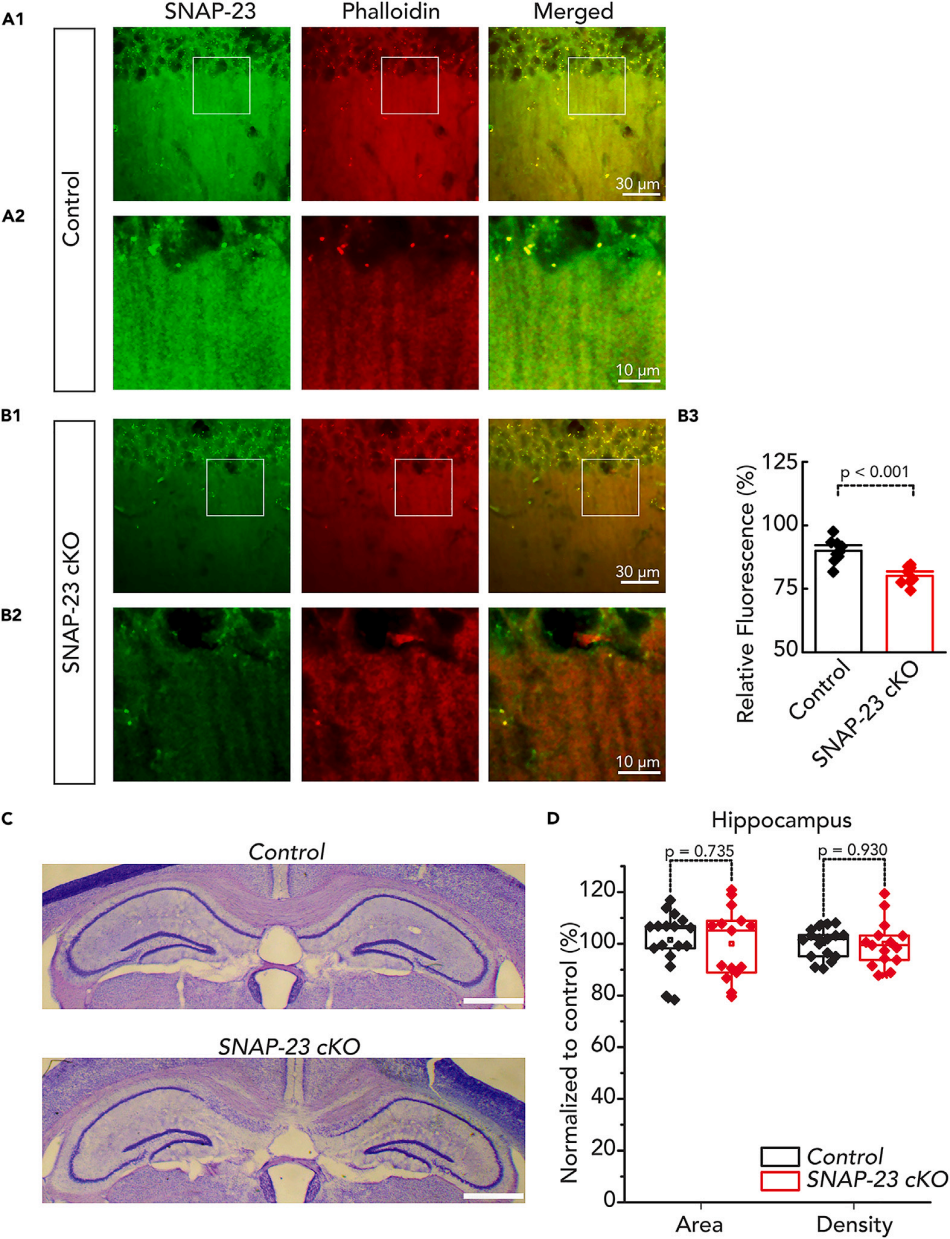
Generation of tissue-specific deletion of SNAP-23 See also Figures S1 and S2. SNAP-23 is specifically removed from CA1 cell dendrites. (A and B) Images obtained from coronal brain sections with the hippocampal CA1 region enlarged. SNAP-23 (green) and phalloidin (red) staining of CA1 dendrites from control (A) and SNAP-23 cKO (B) sections. CA1 dendrite staining (A2, B2) shows a decrease in SNAP-23 cKO slice, quantified in (B3), (independent t-test, t_(16)_ = 5.152, p < 0.001). Scale bar as indicated, 30 μm for panels A1 and B1, 10 μm for panels A2 and B2. (C) Coronal brain sections obtained from control and SNAP-23 cKO mice and stained with cresyl violet; bilateral hippocampal areas enlarged for illustration. Scale bar = 1 mm. (D) Area and density quantification of hippocampi of SNAP-23 cKO mice and control mice. Quantification was done in ImageJ (NIH, Bethesda, Maryland); hippocampi were manually selected, then area and intensity were quantified and normalized to control. n = 17 for control, n = 15 for SNAP-23 cKO. No differences were observed between groups for area (t_(30)_ = 0.342, p = 0.735) or density (t_(30)_ = 0.089, p = 0.930); slices taken from 4 mice each. Error bars represent SEM.

We attempted to confirm the deletion of SNAP-23 protein in pyramidal neurons by immunohistochemistry of hippocampal sections using two-photon microscopy. SNAP-23 is expressed ubiquitously and was seen throughout the examined CA1 hippocampal region. Consistent with previous reports, SNAP-23 immunostaining showed colocalization with postsynaptic phalloidin staining (Figures 1A and 1B).^24^ *Phalloidin* is known to primarily label the periodic actin lattice in dendritic *spines* in acute hippocampal sections.^32^ However, we also saw the fluorescence signal of SNAP-23 outside of phalloidin punctate. We interpreted this as the existence of SNAP-23 in the presynaptic neuron as well. CaMKIIα-Cre is expressed highly in the hippocampus,^26^^,^^33^ as such, we consistently saw a decrease in SNAP-23 signals in the dendritic spines of CA1 neurons in the SNAP-23 cKO sections (Figures 1A and 1B). Fluorescence intensity of postsynaptic SNAP-23 was analyzed using the relative SNAP-23 fluorescence normalized to phalloidin signal. The resulting puncta analysis demonstrated a significant decrease in SNAP-23 signal in the SNAP-23 cKO brains compared to the control (Figure 1B3, independent t-test, t_(16)_, p < 0.001). However, since CaMKIIα-Cre only removes SNAP-23 from pyramidal neurons, glia continues to express SNAP-23. Residual glial SNAP-23 and autofluorescence may contribute to the remaining fluorescence observed. Nevertheless, the gross morphology of the entire hippocampus remained unchanged following SNAP-23 deletion, as identified by cresyl violet staining [n = 17 for control, n = 15 for SNAP-23 cKO, area: t_(30)_ = 0.342, p = 0.735; density: t_(30)_ = 0.089, p = 0.930] (Figures 1C and 1D). We also confirmed that our cKO model is indeed region-specific; SNAP-23 is expressed ubiquitously, and therefore found in the thalamus, however, CaMKIIα-Cre is minimally expressed in the thalamus.^26^ Thus, we imaged the thalamic area to observe SNAP-23 expression. Unsurprisingly, the thalamic area showed no changes to SNAP-23 expression levels following CaMKIIα-Cre-mediated conditional removal (Figure S1) [n = 6 for both groups]. From this, we conclude that our conditional removal is pyramidal neuron-specific and SNAP-23 expression in other regions of the brain is unaffected by CaMKIIα-Cre-mediated removal.

The SNAP family consists of four members, SNAP-23, SNAP-25, SNAP-29, and SNAP-47. SNAP-23 and SNAP-25 are the most similar and share 59% sequence homology,^23^ while SNAP-29 is only 31% similar to SNAP-23.^34^ SNAP-47 is the newest to the family, and its most similar family member is SNAP-29. To rule out the possibility that different SNAP isoforms can compensate in the absence of SNAP-23, we used immunohistology to examine the expression of the different SNAP isoforms following SNAP-23 removal (Figure S2). To do this, we stained brain sections with phalloidin and antibodies against the various SNAP isoforms as before. The relative fluorescence intensity of these SNAP isoforms was examined in the postsynaptic dendritic spine of the CA1 using puncta analysis as before. We found no changes to expression of SNAP-25, SNAP-29 or SNAP-47 following SNAP-23 removal by cKO (independent t-test, SNAP-25: t_(16)_ = −0.900, p = 0.381; SNAP-29: t_(16)_ = −1.153, p = 0.266, SNAP-47: t_(16)_ = −1.374, p = 0.188). Therefore, even though other SNAP family members have proposed roles in postsynaptic receptor trafficking, in the absence of SNAP-23, it is unlikely that a different SNAP family member is upregulated to carry out compensatory effects. We conclude that our SNAP-23 cKO line allows us to examine the functional role of neuronal SNAP-23 in the hippocampus in the absence of major structure abnormality or upregulation of other SNAP isoforms.

### No significant change to evoked fEPSP in tissue-specific SNAP-23 cKO mice

**Figure 2:**
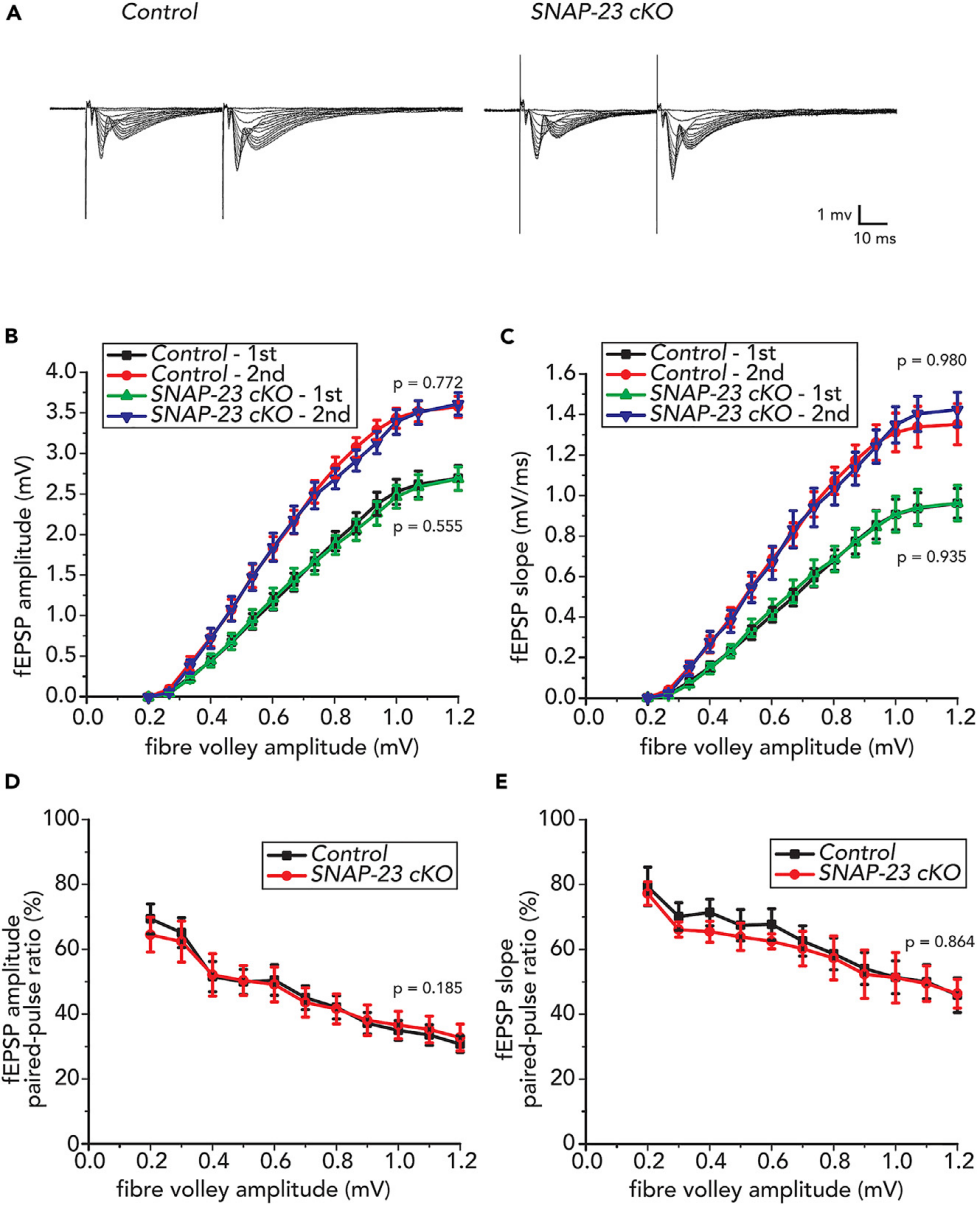
Tissue-specific deletion of SNAP-23 results in no significant changes to fEPSP measures of hippocampal CA1 pyramidal neurons The Schaffer collateral axonal fibers were stimulated twice of 50 ms apart and stimulating intensities varied successively from 10 to 150 μA. The resulting local fEPSP from CA1 pyramidal cell apical dendrites were recorded. (A) Sample traces from control and SNAP-23 cKO mice of dendritic fEPSP. Stimulation artifact truncated for illustrator purposes. (B) fEPSP amplitudes and (C) fEPSP slope were plotted against the presynaptic fiber volley amplitude for control (first stimulation, black; second stimulation, red), and SNAP-23 cKO (first stimulation, green; second stimulation, blue) mice. No difference in amplitude observed between groups for first stimulation [mixed ANOVA: F_(1,47)_ = 0.354, p = 0.555] or second stimulation [F_(1,47)_ = 0.085, p = 0.772]. No difference in slope observed between groups for first stimulation [F_(1,47)_ = 0.001, p = 0.980] or second stimulation [F_(1,47)_ = 0.007, p = 0.935]. (D and E) Paired-pulse ratios of amplitudes (D) or slopes (E) were plotted against presynaptic fiber volley amplitudes. No significant changes between SNAP-23 cKO and controls were observed for paired pulse ration amplitude [F_(1,47)_ = 1.821, p = 0.185] or slope [F_(1,10)_ = 0.030, p = 0.864]. For all measures, control n = 6 mice, 28 slices; SNAP-23 cKO n = 5 mice, 21 slices. Error bars indicate SEM.

We first tested if the CA3-CA1 synapses in SNAP-23 cKO mice exhibit changes in basal neurotransmission by using acute hippocampal slices with standard artificial cerebral spinal fluid (ACSF) perfusion. Field excitatory postsynaptic potentials (fEPSPs) were recorded from the apical dendrites of CA1 pyramidal neurons. A bipolar tungsten stimulating electrode was placed in the *striatum radiatum* of the CA2 region to stimulate Schaffer collateral axons, followed by delivery of paired stimuli of 50 ms apart at incremental intensities from 10 to 150 μA (Figure 2A; see STAR Methods). As more presynaptic axons are recruited with increasing stimulation intensities, we were able to quantify the amplitudes of the presynaptic fiber volley as a proxy of “input.” Input-output curves were generated to compare the fEPSP amplitudes and slopes between SNAP-23 control and cKO groups (Figures 2B and 2C). Increasing the amplitudes of the presynaptic fiber volley resulted in higher amplitudes and slopes of fEPSPs in the CA1 neurons, and such rise in both parameters was similarly observed in the control and the SNAP-23 cKO groups (Figures 2A–2C). In response to the paired stimuli, the ratios of the second vs. first responses in the SNAP-23 cKO group were unchanged from that of the control group (Figures 2D and 2E). Together, these results indicate that SNAP-23 cKO pyramidal neurons result in no evident or significant changes to basal neurotransmission and short-term plasticity.

### Tissue-specific SNAP-23 deletion causes no change in both AMPAR and NMDAR-mediated fEPSPs or single cell EPSCs

**Figure 3:**
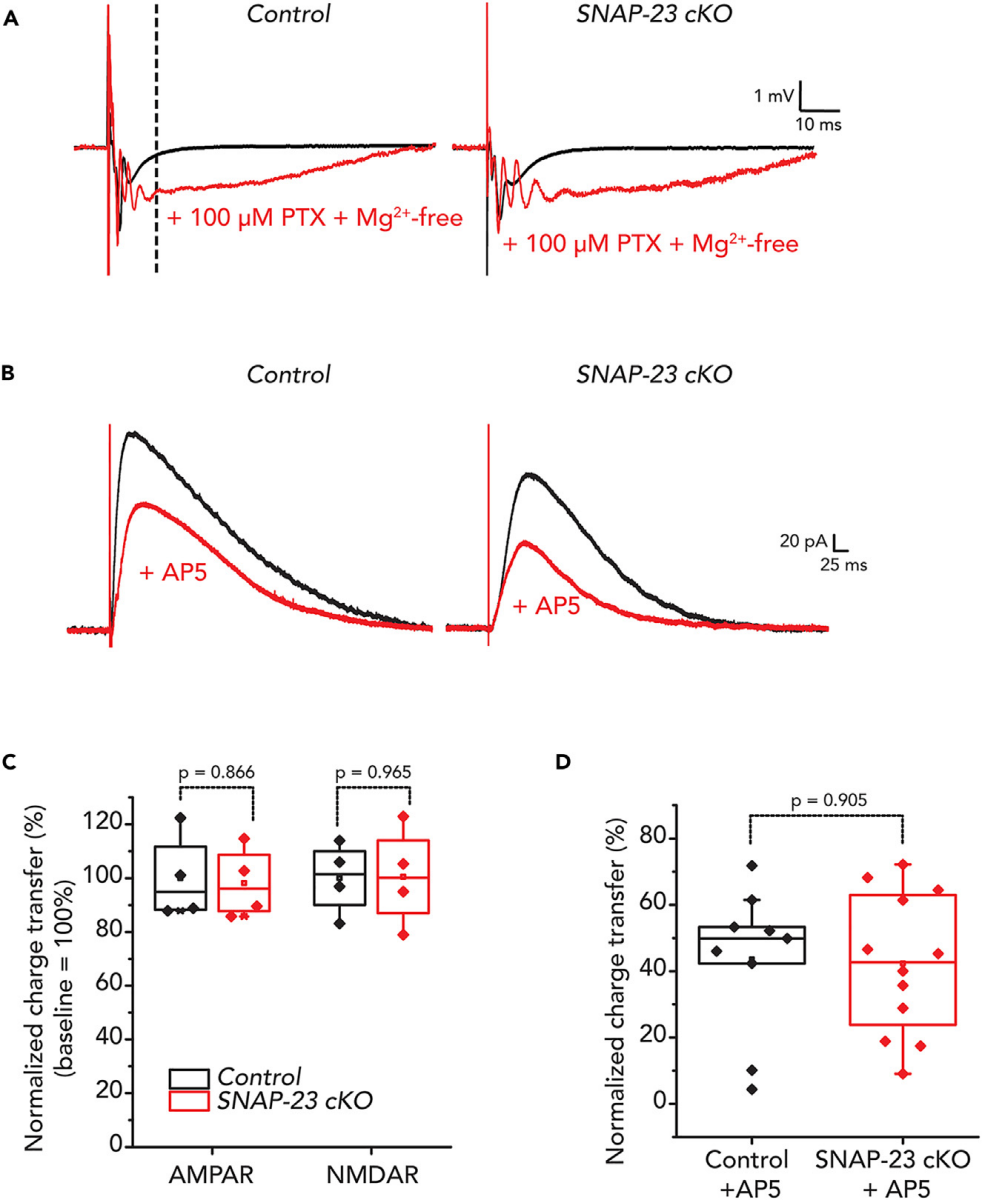
SNAP-23 is dispensable for AMPAR and NMDAR-mediated fEPSP and evoked NMDAR response. See also Figure S3 (A) Dendritic fEPSP recordings from the CA1 area of control and SNAP-23 cKO mice in standard ACSF (black traces) and in Mg^2+^-free plus 100 μM picrotoxin (PTX) ACSF (red traces). Dashed line denotes from where AMPAR current calculation end and NMDAR current calculation begin. Stimulation artifact truncated for illustration purpose. (B) Representative traces of voltage clamp experiments where CA1 pyramidal neurons were voltage clamped at +40 mV to observe both AMPAR and NMDAR-mediated responses. Recordings were done in 100 μM PTX containing ACSF. AP5 was bath applied for 5 min to block the NMDAR component so only the AMPAR-mediated component remains (red). (C) Normalized percent AMPAR and NMDAR-mediated charge transfer in control and SNAP-23 cKO mice. The recordings obtained using Mg2+-free ACSF plus 100 μM PTX were analyzed for this purpose. The area under the curve during this AMPAR activity was calculated as AMPAR-mediated charge transfer while remaining response was calculated as NMDAR-mediated charge transfer. The charge transfer of both AMPAR and NMDAR was then normalized to the control group. Error bars indicate SEM (slice n = 4 for both groups, animal n = 3 for both groups). No significant group differences observed in AMPAR (independent t-test, t_(6)_ = 0.176, p = 0.866) or NMDAR (t_(6)_ = −0.046, p = 0.965) responses. Stimulation artifacts truncated for illustration purpose. (D) Normalized percent NMDAR charge transfer in control and SNAP-23 cKO mice. The area under the curve was calculated, the difference after AP5 application being the NMDAR component. The charge transfer was then normalized to the control group. No difference was observed in the SNAP-23 cKO mice compared to the control mice (t_(19)_ = −0.122, p = 0.905). Error bars indicate SEM.

A previous study using an shRNA-mediated knockdown approach demonstrated that SNAP-23 is involved in the delivery of NMDARs to the postsynaptic membrane.^24^ Therefore, we investigated whether selective changes in AMPAR and/or NMDAR current could be observed using a pharmacological blockade approach in SNAP-23 cKO slices (Figure 3). A stable baseline recording was first obtained in standard ACSF, showing AMPAR-mediated fEPSPs predominantly (Figure 3A, black traces). Switching the bath solution to Mg^2+^-free ACSF containing 100 μM picrotoxin allowed us to observe maximal ionotropic glutamate receptor-mediated fEPSPs by activating NMDAR-mediated responses and by blocking inhibitory GABAergic innervations (Figure 3A, red traces). The evoked response revealed enhanced glutamate receptor-mediated fEPSP with a characteristic prolonged epileptiform response containing multiple spikes in the early phase (Figure 3A, left of dashed line). AMPARs largely mediate the multiple spikes in the early phase of the evoked response while the slow, long-lasting later phase is largely mediated by NMDARs.^28^ Contributions of AMPAR and NMDAR-mediated fEPSPs were quantified by calculating the charge transfer by integrating the area under the corresponding part of the traces.^35^ In contrast to previously reported results,^24^ we found no differences in AMPAR and NMDAR charge transfers in both the SNAP-23 cKO and the control groups (Figure 3C). The ratios of charge transfer from the AMPAR and the NMDAR-mediated responses were also unchanged between the control and the SNAP-23 cKO groups. Therefore, we found that SNAP-23 neither regulates basal AMPA nor NMDA responses in postsynaptic neurons.

To further investigate the effects of SNAP-23 deletion on the AMPA and NMDA currents, we performed whole-cell patch clamp recordings in CA1 neurons of the hippocampal slices (Figure 3B). CA1 neurons were voltage clamped at +40 mV to observe both AMPAR and NMDAR-mediated responses. After a stable baseline was obtained, AP5 was bath applied for 5 min, blocking the NMDAR component so only the AMPAR-mediated component remained. The area under the curve was then calculated, with the difference after AP5 application being the NMDAR component. AP5 application induced a 56.5% decrease in control slices. Similarly, a 57.6% decrease was observed in SNAP-23 cKO slices. Therefore, in agreement with our extracellular recording data, we found that NMDA currents were not changed in SNAP-23 cKO pyramidal neurons relative to controls (Figure 3D). To confirm the residual responses were authentically AMPAR-mediated after the post-AP5 baseline stabilized, we applied 6-cyano-7-nitroquinoxaline-2,3-dione (CNQX; 20 μM) to block any remaining AMPAR-mediated responses. Following the CNQX application, the remaining AMPAR-mediated response was indeed blocked (Figure S3). Together, these data suggest that SNAP-23 does not play a role in the regulation of basal AMPA or NMDA currents.

### SNAP-23 deficient CA1 neurons exhibit normal excitability

**Figure 4:**
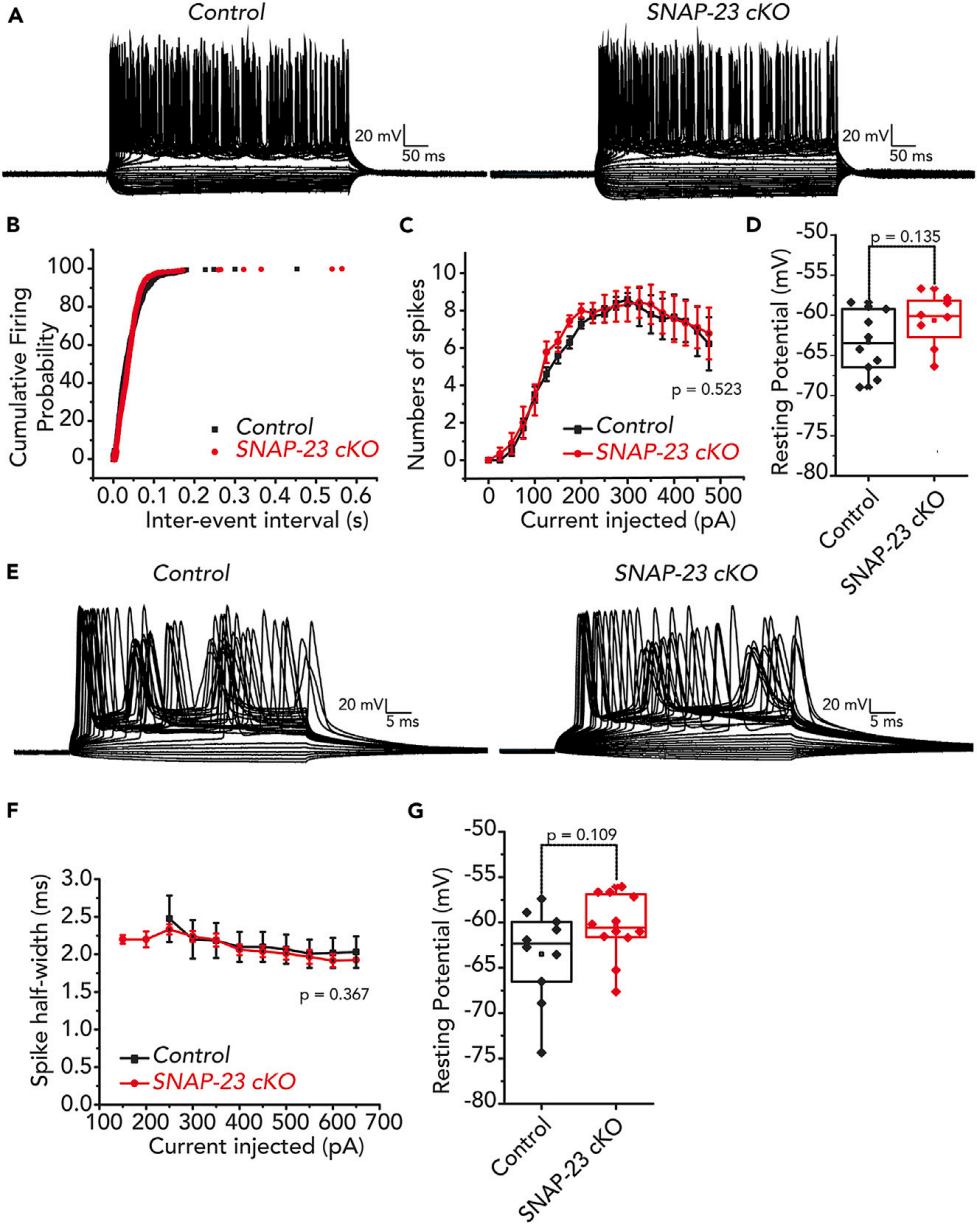
Intrinsic excitability remains the same in CA1 pyramidal neurons of SNAP-23 cKO mice (A–D) Representative traces of current clamp experiments where currents from −300 pA to +500 pA were injected in 25 pA steps and changes in voltages from CA1 neurons of control and SNAP-23 cKO mice were monitored. Intrinsic properties including cumulative firing probability (B), numbers of spikes induced by incremental current intensities (C), and resting membrane potentials (D). No difference were observed in number of spikes [mixed ANOVA: F_(1,16)_ = 0.427, p = 0.523] or membrane potential (independent t-test, t_(16)_ = −1.573, p = 0.135). (E–G) Representative traces of current clamp experiments where currents from −100 pA to +225 pA were injected in 25 pA steps and changes in voltage from CA1 neurons of control and SNAP-23 cKO mice were monitored. Intrinsic properties including spike half-width (F) and resting membrane potential (G). No difference were observed in spike half-width [F_(1,8)_ = 0.913, p = 0.367) or resting membrane potential (t_(20)_ = −1.677, p = 0.109). Intrinsic properties were measured from neurons that had formed series resistance of <20 MΩ for more than 10 min and resting membrane potentials less than −55 mV. Error bars indicate SEM.

We then investigated whether the intrinsic excitability of the CA1 neurons is affected by SNAP-23 deletion. For this, a current clamp was performed where constant square pulses (1 s) of increasing amounts of currents (from −300 pA to +500 pA in 25 pA increments) were injected and changes in voltage responses were monitored to gage the intrinsic excitability of CA1 neurons (Figure 4A). Single spike parameters of CA1 neurons were also measured using current clamp, where shorter constant square pulses (100 ms) of increasing amounts of currents (from −100 to +225 pA in 25 pA increments) were injected and corresponding changes in voltage responses were monitored (Figure 4E). We found that the CA1 neurons of the SNAP-23 cKO mice exhibited similar basal intracellular properties and spiking properties as the control mice. The resting membrane potential, inter-spike intervals, spike half-width, and peak amplitudes were also similar between the two genotypes (Figures 4B–4D, 4F, and 4G).

**Figure 5:**
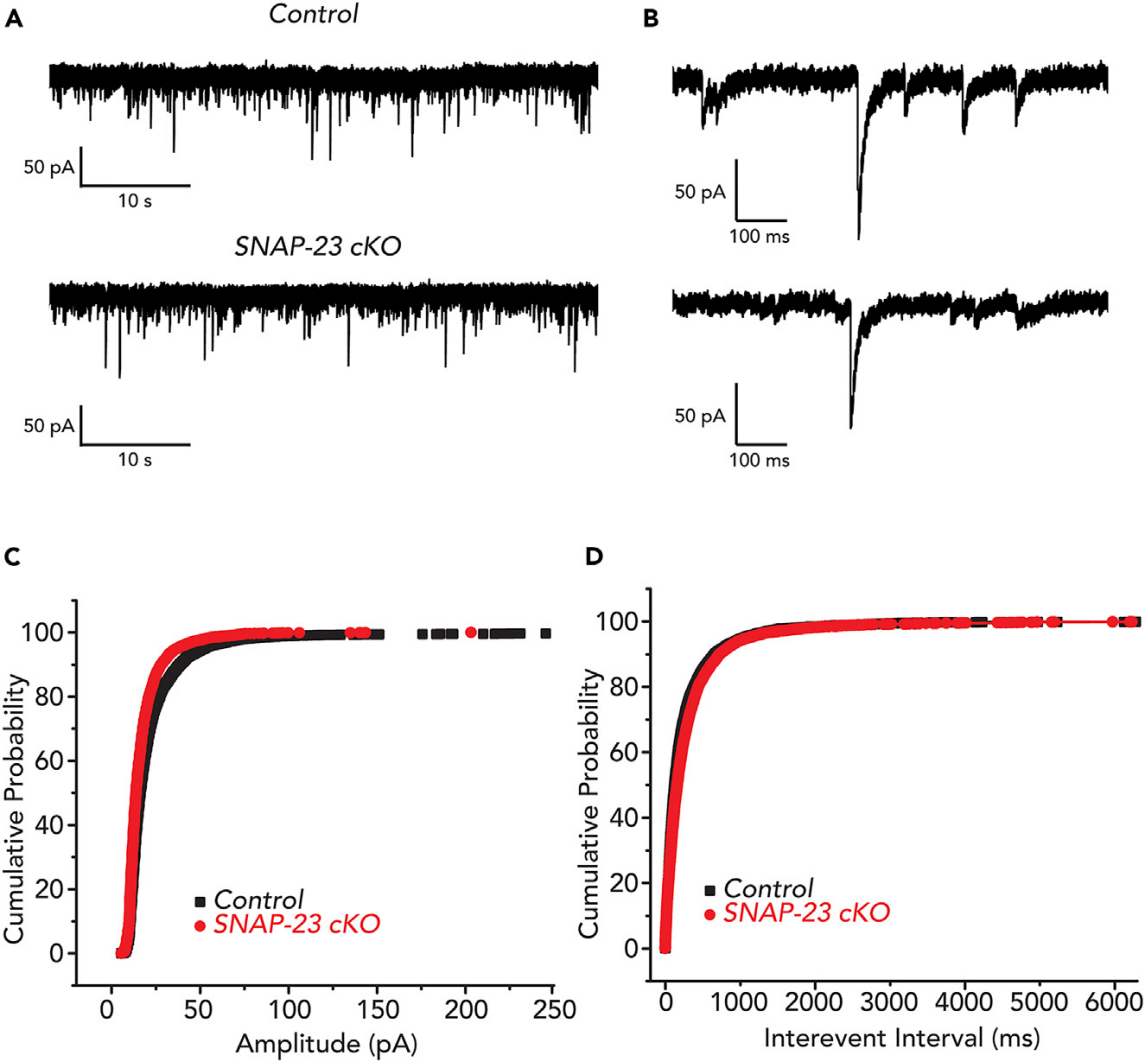
Amplitude and inter-event interval of spontaneous EPSC of CA1 pyramidal neurons are not altered in SNAP-23 cKO mice (A) Representative traces of spontaneous EPSC recorded at −70 mV from CA1 pyramidal neurons of control and SNAP-23 cKO mice. (B–D) Expanded events as denoted in A. Cumulative amplitude (C) and inter-event interval (D) plot of spontaneous EPSCs of control and SNAP-23 cKO. Data were collected from 9 control and 9 cKO CA1 pyramidal neurons. Spontaneous EPSCs were measured from neurons that had formed series resistance of <20 MΩ for more than 10 min.

We then monitored the excitatory postsynaptic currents (EPSCs) from individual CA1 neurons in the acute hippocampal slices. Using a holding potential of −70 mV, we measured the frequencies and amplitudes of spontaneous EPSCs in CA1 neurons (Figure 5). Similar to evoked fEPSPs, we found that the average frequency and amplitude of the spontaneous EPSCs in the CA1 neurons remained unchanged in the SNAP-23 cKO mice (Figure 5D). Collectively, these data demonstrate that SNAP-23 removal does not compromise postsynaptic excitability, as indicated by the unchanged basal properties and excitability observed in the SNAP-23 cKO CA1 neurons.

### SNAP-23 is essential for long-term potentiation

Having established that the SNAP-23 deletion does not affect basal hippocampal transmission, we next investigated the role of SNAP-23 in synaptic plasticity. Specifically, we tested whether SNAP-23 deletion affects LTP, a major form of synaptic plasticity and a local circuitry correlate of learning and memory. For this purpose, we prepared acute hippocampal slices and measured the apical dendritic fEPSPs from CA1, before and after inducing LTP. We chose to use the theta-burst stimulation protocol, as it is a more physiologically relevant LTP protocol compared to high-frequency stimulation. We gave 3 s of continuous theta-burst stimulation: 15 bursts of four pulses at 100 Hz, with an interburst interval of 200 ms in Schaffer collateral axons. For each slice, the stimulation intensity was chosen such that it elicited 30% of the maximum response. fEPSP were evoked every 30 s before and after theta burst stimulation and the onset slopes of evoked fEPSPs were measured.

**Figure 6:**
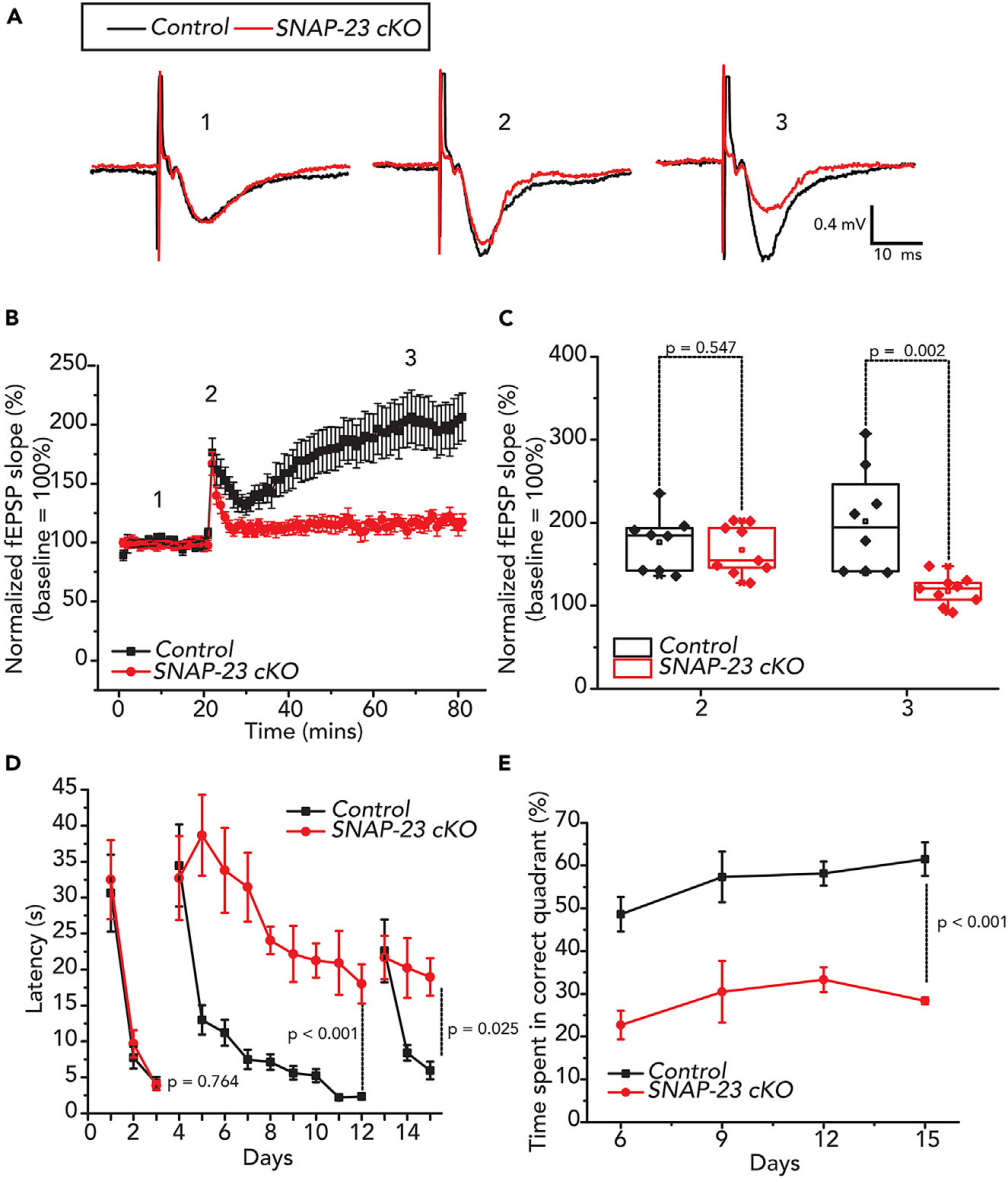
CA1-specific SNAP-23 cKO results in defective Schaffer collateral-CA1 LTP and significantly impairs Morris water maze performance. See also Figure S4 (A) Representative recordings of fEPSP of control and SNAP-23 cKO CA1 at baseline (1), immediately after LTP induction (2), and 50 min after LTP induction (3); black traces control, red traces SNAP-23 cKO. Each illustrated trace was averaged from four consecutively evoked responses. (B) Average fEPSP slope normalized against baseline. Data were collected from 9 slices of KO mice and 8 slices of control mice. (C) Normalized fEPSP slope for control and SNAP-23 cKO at times 2 (immediately after LTP induction) and 3 (50 min after LTP induction). Error bars indicate SEM (independent t-test, t_(6)_ = 0.617, p = 0.547 for timepoint 2, t_(15)_ = 3.835, p = 0.002 for timepoint 3). (D) Learning curves for mice to find the visible platform (days 1–3), hidden platform (days 4–12) and hidden platform during reversal training (days 13–15). Animals were tried 4 times a day, with randomized entry points to the pool. Latency to find the hidden platform was significantly increased in the SNAP-23 cKO mice relative to the controls [mixed ANOVA; days 4–12: F_(1,10)_ = 101.9, p < 0.001; days 13–15: F_(1,10)_ = 6.924, p = 0.025], however, SNAP-23 cKO mice still demonstrate some degree of learning [F_(8,10)_ = 14.752, p < 0.001]. (E) During Probe Test, platform was removed from the pool and % of time spent in quadrant where platform was previously located was calculated from total recording time of 60 s, SNAP-23 cKO mice spent less time in the correct quadrant during the probe test [F_(1,10)_ = 55.420, p < 0.001]. Error bars indicate SEM.

We examined CA1 LTP induction in 9 slices of 5 SNAP-23 cKO mice and 8 slices from 4 control mice. Following the theta-burst stimulation delivery, post-tetanic potentiation (PTP) amplitudes and slopes at more than 150% of the baseline were observed for both control and the SNAP-23 cKO groups (Figures 6A–6C). However, there were no group differences in PTP measures [independent t-test, t_(15)_ = 0.617, p = 0.547; Figure 6C]. In the control group, the PTP response transitioned to an evident LTP phase, which stabilized at more than ∼150% of baseline levels and lasted as long as 60 min after the theta-burst stimulation (Figures 6A–6C). In contrast, the SNAP-23 cKO group showed a weak LTP phase, which was maintained at levels less than 125% of baseline responses and was significantly diminished from the control [t_(15)_ = 3.83, p = 0.002]. Prior to LTP, fEPSPs were similar between the SNAP-23 cKO and control groups. Analysis of theta burst induction also showed no group differences (Figures S4A and S4B), [mixed ANOVA, stimulation 1: F_(1, 21)_ = 0.91, p = 0.766, stimulation 2: F_(1, 21)_ = 0.608, p = 0.444, stimulation 3: F_(1, 21)_ = 0.835, p = 0.371], further supporting that there is no significant difference in the induction phase of LTP. However, when we analyzed the fEPSP slope 0.5 to 5 min after LTP induction, we found that the post-theta stimulation phase of the LTP response was decreased in the SNAP-23 cKO group [F_(1, 15)_ = 7.072, p = 0.018] (Figure S4C). Therefore, while CA1 LTP is greatly compromised in SNAP-23 cKO slices, we do not exclude presynaptic or other changes to our SNAP-23 cKO phenotype. These observations, together with the previous assessments of CA1 evoked fEPSPs (Figure 2), indicate that SNAP-23 is selectively critical for synaptic plasticity without affecting basal transmission.

### Tissue-specific SNAP-23 KO mice exhibit impaired spatial memory

We next investigated the behavioral consequences of LTP deficits seen in the SNAP-23 cKO mice by performing the Morris water maze to assess spatial learning and memory.^36^^,^^37^ A 15-day protocol was performed wherein each day was composed of four separate trials. During the four trials, the mice were placed into the pool from a randomized cardinal position (Figures 6D and 6E). Throughout the first three days, a flag was present on the platform as a visible cue to allow mice to learn the cued task. From day 4 onwards, the flag was removed, the position of the hidden platform was changed, and the mice were asked to learn the position of the hidden platform. The SNAP-23 cKO group (n = 6) effectively learned the visible cued swimming task as their latency to find the platform followed a similar reduction pattern to the control group (n = 6) by day 3. There was no statistical difference between the two groups [mixed ANOVA: F_(1,10)_ = 0.095, p = 0.764], indicating that the SNAP-23 cKO did not impair the ability to visualize surroundings and swim (Figures 6D and 6E). The control animals had a steep drop in the latency to find the platform between days 4 and 5, which is indicative of quickly learning the new location of the platform and demonstrating effective spatial learning. On the other hand, the SNAP-23 cKO mice displayed impaired spatial learning and memory as they needed significantly more time to find the hidden platform in later days. Mixed ANOVA showed that there is a statistically significant difference between the two groups [F_(1,10)_ = 101.9, p < 0.001]. Nonetheless, SNAP-23 cKO did exhibit some learning capacity as the latency decreased by day 12 of the trial [effects of days: F_(8,10)_ = 14.752, p < 0.001], but this learning was at a significantly slower rate than that of the littermate controls [interactions between groups and days: F_(1,8)_ = 3.992, p < 0.001]. SNAP-23 cKO mice also spent less time in the correct quadrant during probe tests (Figure 6E), further demonstrating the impairment to spatial learning arising from SNAP-23 deficiency [F_(1,10)_ = 55.420, p < 0.001]. On day 13, a reversal training was instated, and the location of the hidden platform was again changed. Both the control and SNAP-23 cKO groups needed more time to find the platform. However, the control group was able to re-learn the location more quickly than the SNAP-23 cKO group [F_(1,10)_ = 6.924, p = 0.025]. Together, our results indicate that SNAP-23 in the CA1 neurons of the hippocampus is critical for spatial memory.

### The behavioral abnormality of SNAP-23 cKO mice is more selective than that of syntaxin-4 cKO mice

**Figure 7:**
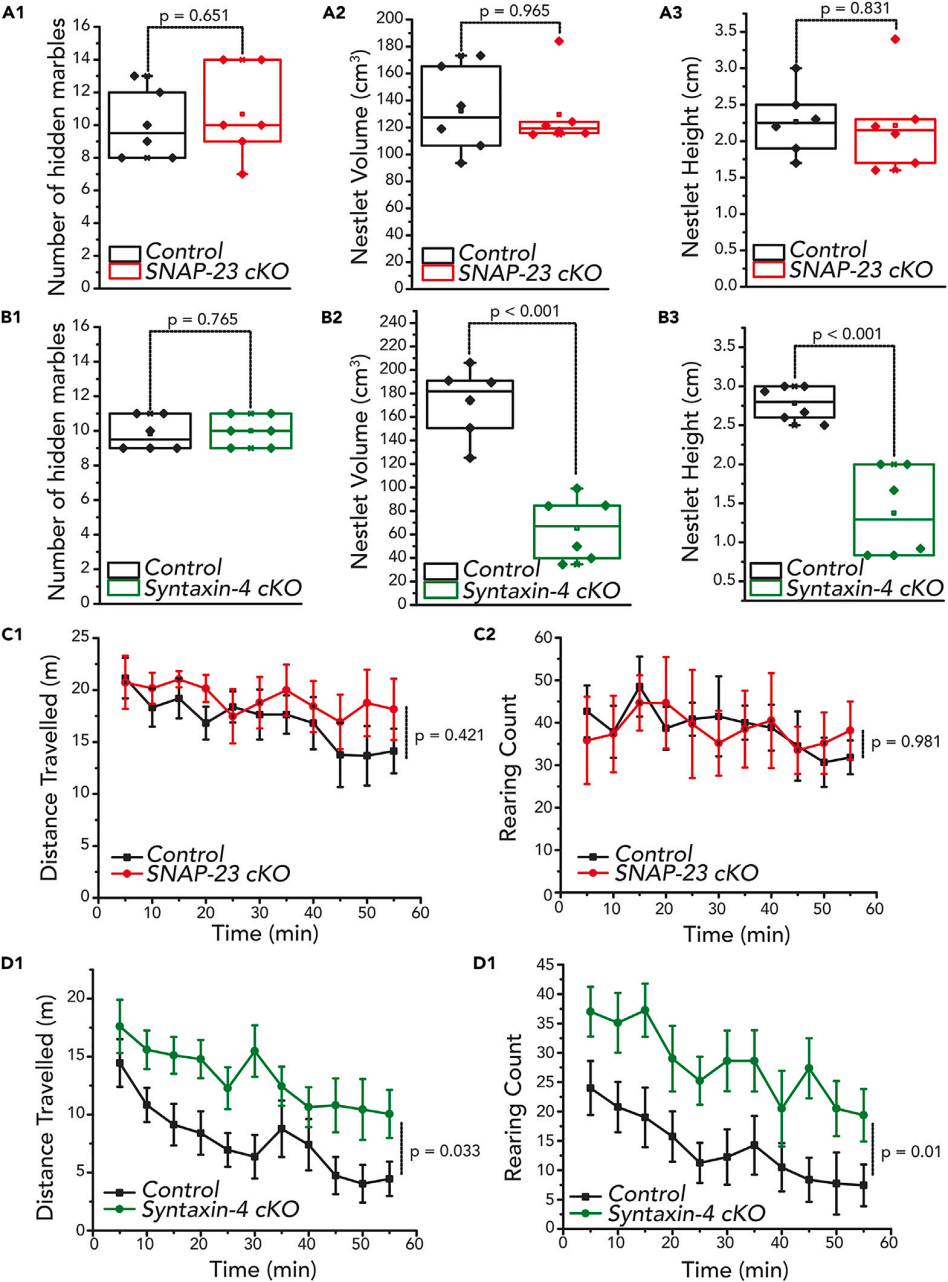
SNAP-23 cKO impairment is memory specific leaving other behaviors intact while Syntaxin-4 cKO impairs other behaviors such as nestlet shredding (A–D) Quantifications of marbles buried and nestlet shredded by SNAP-23 cKO (A) and syntaxin-4 cKO mice (B); both mice buried a comparable number of marbles when compared to control (independent t-test, t_(10)_ = −0.466, p = 0.651 for SNAP-23 cKO; t_(10)_ = 0.307, p = 0.765 for syntaxin-4 cKO). However, syntaxin-4 cKO mice shredded less amount of nestlet compared to control (t_(10)_ = 5.586, p = 0.001 for nestlet height; t_(10)_ = 6.500, p = 0.001 for nestlet volume) while SNAP-23 shredded amounts similar to that of control (t_(8)_ = 0.221, p = 0.830 for nestlet height; t_(8)_ = −0.456, p = 0.965 for nestlet volume). Open field test of SNAP-23 (C) and syntaxin-4 (D) mice, distance traveled and rearing count were assessed. SNAP-23 cKO mice show no changes to both distance traveled [mixed ANOVA: F_(1,10)_ = 0.703, p = 0.421] or rearing count [F_(1,10)_ = 0.001, p = 0.981] when compared to littermate controls, while syntaxin-4 cKO mice show an increase to spontaneous locomotion [F_(1,14)_ = 5.567, p = 0.033] and rearing count [F_(1,14)_ = 8.739, p = 0.010] when compared to littermate controls. Error bars denote SEM.

We previously found that in syntaxin-4 cKO mice, basal transmission, as well as LTP, were both strongly decreased,^28^ which supports previous findings that syntaxin-4 directs synaptic plasticity.^38^ Similar to the SNAP-23 cKO, the syntaxin-4 cKO mice showed deficits in their Morris water maze performances.^28^ Here, we investigated other sets of behavior tasks using the SNAP-23 cKO and the syntaxin-4 cKO mice to uncover any correlations between synaptic and behavioral abnormalities. We first performed the marble burying test and nest shredding test (Figures 7A and 7B). Marble burying and nestlet shredding are tasks often used to gain insight into repetitive behaviors; moreover, nestlet shredding is a task that involves the hippocampus.^39^ We found that the marble burying was unchanged in the syntaxin-4 cKO mice (n = 6) compared to the littermate controls (n = 6) [independent t-test, t_(10)_ = −0.307, p = 0.765]. However, the syntaxin-4 cKO mice showed differences in the nestlet shredding task, completing the test with less amounts of shredded nestlet [height t_(10)_ = 5.586, p = 0.001; volume t_(10)_ = 6.500, p = 0.001]. In contrast, there was no change to the level of repetitive behaviors of the SNAP-23 cKO mice in both tests (Figure 7A).

We also performed an open-field test to assess changes in the level of anxiety in these animals. Here, we evaluated spontaneous locomotor activity and the rearing pattern of each genotype under a novel environment (Figures 7C and 7D). We found that syntaxin-4 cKO mice displayed striking increases in spontaneous locomotion and rearing behavior, as compared with littermate control mice. However, we did not find differences in spontaneous locomotion or rearing behavior between SNAP-23 cKO and the littermate control group. Together, these results suggest that changes in basal neurotransmission seem to affect repetitive and spontaneous locomotor activity. However, LTP is more specifically required for learning and memory wherein SNAP-23 plays a crucial role.

## Discussion

By generating a pyramidal neuron-specific knockout of SNAP-23, we were able to demonstrate the role of neuronal SNAP-23 in basal synaptic transmission and synaptic plasticity as well as its behavioral consequences. Deletion of SNAP-23 did not show major abnormalities to the morphology of the hippocampus (Figure 1), basal synaptic transmission (Figure 2) including AMPAR and NMDAR-mediated postsynaptic currents (Figures 3 and 5), intrinsic excitability of CA1 pyramidal neurons (Figure 4) or repetitive and spontaneous locomotive behavior (Figure 7). However, SNAP-23 cKO impaired LTP and spatial memory (Figure 6). Therefore, we conclude that neuronal SNAP-23 is selectively crucial for synaptic plasticity and spatial memory.

Studying the mechanisms of receptor trafficking is difficult since neurons express multiple isoforms, including the neuronal SNAP-25, but also SNAP-23,^19^^,^^24^^,^^40^ SNAP-29,^41^ and SNAP-47.^22^^,^^42^^,^^43^ The concurrent expression of these isoforms may suggest distinct roles; however, it also introduces a confound wherein the absence of one protein may cause one or more of the other isoforms to compensate for the loss. Indeed, there is evidence suggesting that SNAP-47 can substitute for SNAP-25 in cultured neurons, albeit with less efficacy.^43^ As such, previous publications have reported highly contradicting results. Such lack of consensus may also be due to differences in methodologies used to study these proteins, as lethality is observed in both SNAP-25^44^ and SNAP-23^30^ global knockout mice. In fact, even a broader and milder nervous system cKO of SNAP-23 using Nestin-Cre, expressed in neuronal and glial cell precursors, resulted in severe phenotypes with animals dying within 3 weeks of birth.^45^ These Nestin-Cre-induced SNAP-23 knockout mice lack hippocampi, further emphasizing the importance of SNAP-23 in brain development. Therefore, many studies on SNAP-23 and other SNAP isoforms have largely relied on transient shRNA knockdown^24^^,^^25^^,^^46^ and heterozygote^24^ approaches. SNAP-47 has been suggested to be involved in AMPAR trafficking,^25^ whereas SNAP-23 and SNAP-25 have been suggested to play a role in NMDAR trafficking.^24^^,^^47^ Conversely, one group found that SNAP-23, not SNAP-25, is responsible for regulating NMDAR expression.^24^ However, another group reported different results and found that SNAP-25, not SNAP-23, was crucial for AMPAR trafficking during LTP, using shRNA knockdown.^25^ This study also found that SNAP-25, not SNAP-23, regulates surface NMDA receptor levels, and the reduction of SNAP-23 fails to impair NMDAR-dependent chemical LTP.^25^ However, a later review brings up the key point that SNAP-23 may play more of a developmental role, and/or residual SNAP-23 from the incomplete knockdown may be sufficient in supporting LTP.^41^ Finally, another group described SNAP-25, but not SNAP-23, as being responsible for enhancing NMDA currents by NMDAR delivery.^46^ While shRNA knockdowns were indeed able to show changes in glutamate receptor trafficking,^24^ or changes to LTP in the case of SNAP-47,^25^ studying the behavioral consequences of these findings is a challenging due to the lack of viable animal models.

With pyramidal neuronal-specific SNAP-23 cKO mice, our data provide evidence that SNAP-23 does not appear to be required for supporting baseline AMPAR and NMDAR responses in CA1 pyramidal neurons but selectively mediates LTP. Previously, studies suggested that SNAP-47 plays this role.^25^ The discrepancies between our current data and previously reported results^24^^,^^25^ can be caused by various reasons, such as chronic SNAP-23 deletion causing compensation by other isoforms, or transient knockdowns insufficiently removing the protein of interest. Moreover, non-specific targeting of shRNA can also contribute to such contradicting results. However, as we observed in our model, despite the chronic removal of SNAP-23 not affecting AMPAR and NMDAR currents, it was able to impair LTP and spatial memory. Here, we were able to show for the first time, behavioral consequences arising from the removal of SNAP-23. As these impairments to LTP and spatial memory were observed in adult mice, it also challenges the notion that SNAP-23 does not play a role in supporting NMDAR currents in adulthood.^41^ Using our SNAP-23 cKO mouse model, we were also able to show that other SNAP isoforms are not upregulated to compensate for the absence of SNAP-23 (Figure S2).

Our data suggest that SNAP-23 carries out a specific role in LTP, and its deletion leaves basal neurotransmission and short-term plasticity largely intact. Presumably, another SNAP isoform carries out functions in basal AMPAR and NMDAR trafficking events. The compatibility of different SNAP proteins in forming differential fusion complexes may lie in the structure of the SNAP protein in question or with its interaction partners. SNARE members are highly promiscuous^48^^,^^49^ and are capable of substituting for each other *in vitro.*^22^ Therefore, this promiscuity begs the question of whether a different SNAP isoform with alternative functions, such as roles in basal AMPAR and NMDAR trafficking, can carry out roles to support LTP. SNAP proteins contribute two α-helices to the four-helix bundle that comprises the SNARE complex. Although SNAP isoforms are structurally similar, which might cf. the promiscuous nature of SNARE proteins, each isoform may still attribute a specific function. A supporting example is that the SNAP-25 N-terminus cannot substitute for the SNAP-47 N-terminus.^25^ Aside from differences between the SNARE motifs, membrane association can also be an attributing factor to the differential roles carried out by these SNAP proteins. SNAP-23 and SNAP-25 are membrane-anchored by palmitoylation, while SNAP-29 and SNAP-47 are not membrane-anchored.^23^ There is a possibility that membrane association confers some of the characteristics that give rise to these differential functions, as indeed SNAP-23 and SNAP-25 can both rescue synaptic vesicle fusion deficits observed in SNAP-25 knockout culture, while SNAP-29 and SNAP-47 cannot.^50^ However, this notion remains obscure as SNAP-23 and SNAP-29 can support dense core vesicle fusion in culture, while SNAP-47 cannot.^50^ Additionally, although SNARE proteins interact with each other promiscuously, not all combinations are fusogenic,^49^ and additional mechanisms may regulate these vesicle fusion processes.

Unlike what we previously observed in syntaxin-4 cKO mice, in which both basal neurotransmission and LTP were diminished, SNAP-23 cKO mice are only impaired in LTP while basal neurotransmission remains intact. While syntaxin-4 cKO mice exhibit abnormalities in various behaviors, deficits of SNAP-23 cKO are selectively limited to spatial memory. Syntaxin-4 cKO mice showed abnormalities in the nestlet shredding test, which contrasts with SNAP-23 cKO mice, where the performance of nestlet shredding was comparable to that of the control. This suggests that the LTP deficits are spatial learning and memory specific, as demonstrated by poor Morris water maze performances, while deficits to the basal neurotransmission may affect other behaviors such as the hippocampus-dependent nestlet shredding task. The observed SNAP-23 cKO phenotypes are also different from syntaxin-3 cKO, as syntaxin-3 cKO displayed unchanged LTP and basal neurotransmission, along with no changes to learning and memory.^33^ This suggests that the regulation of glutamate receptor trafficking in postsynaptic membranes is highly heterogeneous, and the requirements of specific SNARE proteins may depend on the neuronal activity state.

**Figure 8:**
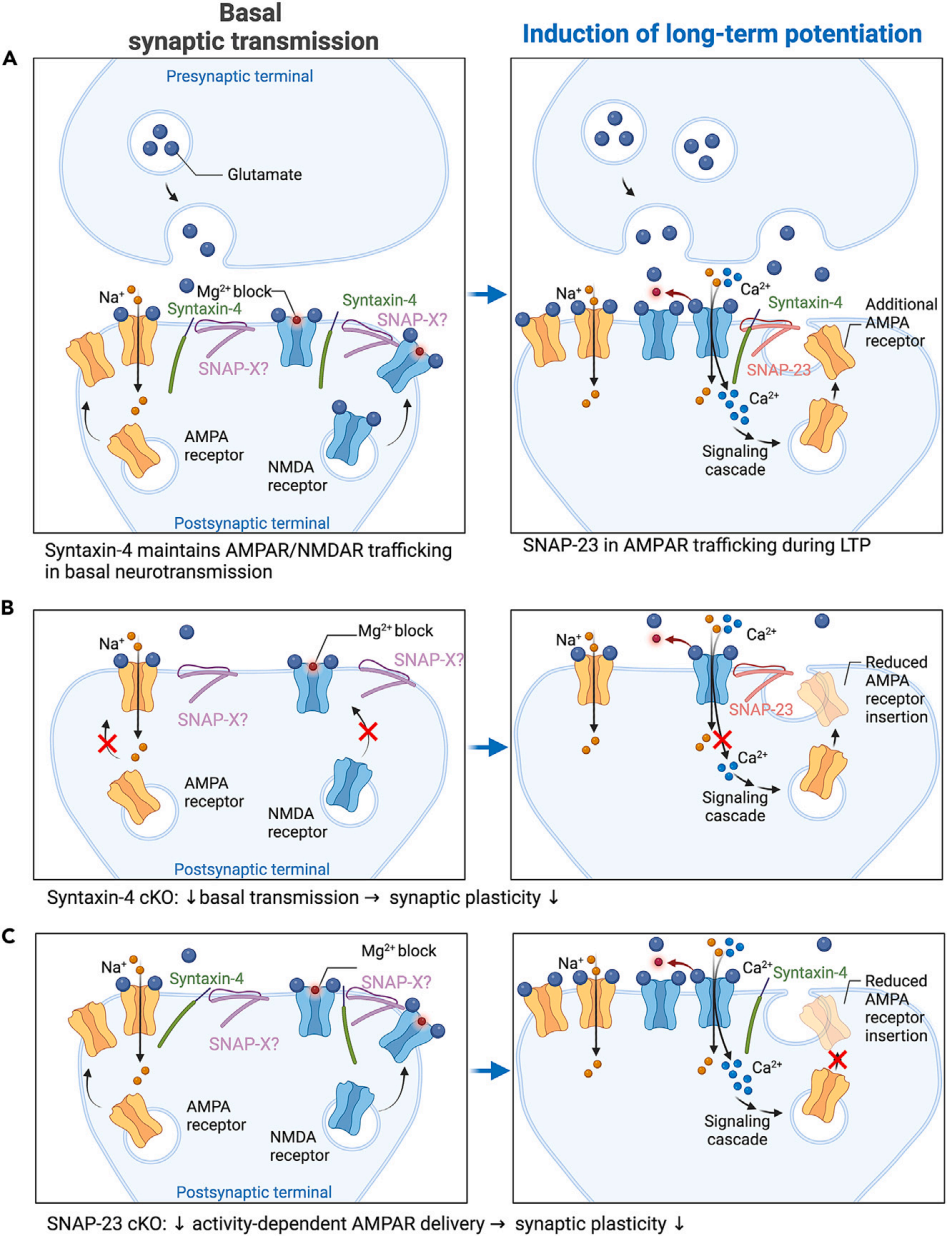
Model of SNAREs involved in basal transmission and LTP (A) Left panels indicate the presynaptic and postsynaptic terminal under basal synaptic transmission conditions. t-SNAREs syntaxin-4 and an unidentified SNAP isoform (SNAP-X) maintain AMPAR and NMDAR levels under basal synaptic transmission conditions. Right panel indicates synaptic changes following induction of long-term potentiation (LTP); following removal of the magnesium block by theta burst stimulation, calcium influx activates signaling cascades to trigger activity-dependent AMPAR insertion. Activity dependent exocytosis requires syntaxin-4 complexing with SNAP-23 to deliver AMPARs during LTP. (B) Syntaxin-4 cKO decreases basal AMPAR and NMDAR numbers and decreases AMPAR insertion during LTP. Also possible is an indirect effect of syntaxin-4 cKO decreases basal NMDAR levels, allowing for less calcium influx during LTP, and reducing AMPAR insertion. (C) SNAP-23 cKO does not alter basal neurotransmission, and acts downstream of calcium signaling cascade, reducing AMPA receptor insertion during LTP. Adapted from “Long-Term Potentiation”, by BioRender.com (2022). Retrieved from https://app.biorender.com/biorender-templates.

Previously, we proposed that syntaxin-4 may play a direct role in ionotropic glutamate receptor trafficking and is responsible for maintaining basal neurotransmission, synaptic plasticity, and hippocampus-dependent learning; syntaxin-4 cKO leads to decreased basal ionotropic glutamate receptor levels and AMPAR insertion during LTP, thereby impairing basal neurotransmission and LTP.^28^ We also speculate that syntaxin-4 exerts an indirect effect – perhaps the decrease in basal NMDAR current, observed in syntaxin-4 cKO, results in decreased calcium influx, preventing sufficient activation of the signaling cascade that induces AMPAR insertion during LTP. In this model, activity-dependent AMPAR trafficking would depend on the t-SNAREs syntaxin-4^28^ and SNAP-23, which we elucidated in this study, albeit through different mechanisms of action (Figure 8). Under this model, SNAP-23 cKO does not alter basal neurotransmission and only alters activity-dependent AMPAR delivery downstream of the calcium signaling cascade, resulting in deficits to synaptic plasticity.

In summary, for the first time, our SNAP-23 cKO mice allowed us to test the role of SNAP-23 *in vivo* and *in vitro*. CKO mice survived to adulthood, enabling spatial learning and memory tests to be performed and opening up new possibilities for an Alzheimer’s disease model.^51^ Our results using the Morris water maze task indicate that the reduction of LTP maintenance is correlated with deficits in spatial learning and memory (Figure 6). However, AMPAR and NMDAR currents, along with NMDA evoked responses, were not reduced, therefore suggesting a dispensable role of SNAP-23 in glutamate receptor trafficking during basal neurotransmission.

### Limitations of the study

While the LTP is considered primarily due to postsynaptic changes (eg. insertion of AMPA receptors),^52^^,^^53^ it can also involve presynaptic changes (eg. enhanced glutamate release,^54^^,^^55^ also reviewed by Nicoll (2017)^56^). Therefore, one limitation of our study is that our results do not exclude such a role of presynaptic SNAP-23 in LTP, which may contribute to the group difference seen in Figure 6. While we found that SNAP-23 removal altered LTP, this alteration could partly be due to the disruption of the presynaptic SNAP-23. Such presynaptic disruption might be primarily relevant to the LTP maintenance phase since the baseline evoked fEPSPs and spontaneous EPSCs were preserved in CA1 neurons of SNAP-23 cKO mice. Therefore, it will be of great interest for future studies to examine the contributions of presynaptic SNAP-23 during LTP.

## STAR★Methods

### Key resources table

**Table:** 

REAGENT or RESOURCE	SOURCE	IDENTIFIER
**Antibodies**		

Rabbit monoclonal anti-SNAP-23	Novus Biologicals	Cat #. NBP2-67157; RRID:AB_2809999
Mouse monoclonal anti-SNAP-25	Covance	SMI-81R; RRID:AB_510034
Rabbit Polyclonal Anti-SNAP-29	Proteintech	12704-1-AP; RRID:AB_2192340
Rabbit Polyclonal Anti-SNAP-47	Synaptic Systems	Cat #. 111 403; RRID:AB_887899
Alexa Fluor 488 Goat anti-Rabbit IgG	Invitrogen	A-11008; RRID:AB_143165
Alexa Fluor 488 Goat anti-Mouse IgG	Invitrogen	A-11001; RRID:AB_2534069

**Chemicals, peptides, and recombinant proteins**		

Taq DNA polymerase	Bio-Helix	Cat #. MB101-0500
Euthanyl	Bimeda-MTC	DIN 00141704
Paraformaldehyde	EM Science	CAS 30525-89-4
PBS Tablets	BioShop	Cat #. PBS404.200
Tissue-Tek O.C.T. Compound	Sakura	Cat #. 62550-12
Triton X-100	Sigma	CAS 9002-93-1
Goat Serum	Gibco	Cat #. 16210-072
Rhodamine Phalloidin	Abcam	Ab235138
Mowiol 4-88	Sigma	Cat #. 81381-250
10% Neutral buffered formalin	Sigma	Cat #. HT501128-4L
Cresyl Violet	Sigma	C5042-10G
Sucrose	BioShop	CAS 57-50-1
Potassium Chloride	BioShop	CAS 7447-40-7
Monosodium phosphate	Sigma	S9638-500G
Glucose	Sigma	G8270-10 KG
Calcium Chloride	BioShop	CCL302.500
Magnesium Chloride	BioShop	MAG510.1
HEPES	BioShop	HEP001.250
Sodium Chloride	BioShop	SOD002.205
Sodium Bicarbonate	BioShop	SOB308.5
Magnesium Sulfate	BioShop	MAG513.500
Potassium D-Gluconate	Fluka	Cat #. 60245
HEPES	Fluka	Cat #. 54459
EGTA	Fluka	Cat #. 03778
NaGTP	Sigma	G8877-100 MG
MgATP	Sigma	A-9187
Phosphocreatine	EMD Millipore	2380-5 GM
TEACl	Sigma	T-2265
AP5	Cayman Chemical Company	CAS 76326-31-3
Picrotoxin	Cayman Chemical Company	Cat #. 20771
CNQX	Tocris	Cat #. 1045

**Experimental models: Organisms/strains**		

Mouse: SNAP23^tm1Jpes^ (SNAP-23 flox)	OZgene, Feng et al.^27^	N/A
Mouse: B6.Cg-Tg(Camk2a-cre)T29-1Stl/J	Jackson Laboratories	#005359 RRID:IMSR_JAX:005359
Mouse: SNAP23^tm1Jpes^; B6.Cg-Tg(Camk2a-cre)T29-1Stl/J (SNAP-23 cKO)	This paper	N/A MGI:6256731

**Oligonucleotides**		

5′- GGGGGTGAGTTGAAGTCATTGAAG-3′	This paper	Primer 1 (Forward)
5′- AGCTTAAACGGGATGAACTCAGGC-3′	This paper	Primer 2 (Reverse)
5′- CCCCAAGCTCGTCAGTCAA-3′	This paper	Primer 3 (Forward)
5′- ACAGAAGCATTTTCCAGGTATGCT-3′	This paper	Primer 4 (Reverse)

**Software and algorithms**		

Adobe Photoshop	Adobe	www.adobe.com
Adobe Illustrator	Adobe	www.adobe.com
CorelDRAW X7	Corel Corporation	www.coreldraw.com
ImageJ	National Institute of Health	imagej.net/software/fiji
Imaris	Oxford Instruments	imaris.oxinst.com
Origin Pro 2016	OriginLab	www.originlab.com/origin
SPSS	International Business Machines Corporation	www.ibm.com/spss
Clampex 9.2	Molecular Devices	www.moleculardevices.com
MultiClamp 2.1.0.16	Molecular Devices	www.moleculardevices.com
Clampfit 9.2.1.9	Axon Instruments	www.moleculardevices.com
MiniAnalysis	Synaptosoft	synaptosoft.com/MiniAnalysis

**Other**		

Glass capillary	World Precision Instruments	TW150F-4
Superfrost Plus Slides	Fisherbrand	Cat #. 12-550-15
Microscope Cover Glass	Fisherbrand	Cat #. 12545M

### Resource availability

#### Lead contact

Further information and requests for resources and reagents should be directed to and will be fulfilled by the lead contact, Shuzo Sugita (shuzo.sugita@uhnresearch.ca).

#### Materials availability

All reagents and mouse lines generated by this study are available from the lead contact.

### Experimental model and subject details

#### Animals

SNAP-23 flox/flox mice were previously created by OzGene by introducing a loxP site flanking exons 3 and 427. C57/B6 mice CaMKIIα-Cre were purchased from Jackson Laboratory (005359). SNAP-23 flox/flox mice were crossed with CaMKIIα-Cre to generate tissue-specific SNAP-23 conditional KO mice. For each animal, genomic DNA was extracted from the tail for genotyping by PCR. SNAP-23 conditional KO mice were viable, fertile and developed to adulthood without obvious behavioral abnormalities or welfare concerns. SNAP-23 flox/flox mice without CaMKIIα-Cre were used as control mice for all experiments. One possible caveat of our model is that although CaMKIIα-cre is generally accepted as a forebrain specific cre-recombinase line, it has also been previously shown that CaMKIIα-Cre can be minimally expressed in the male testis.^57^ This introduces a small possibility of a global ablation to SNAP-23. To mitigate this confound, breeding pairs were set up so the CaMKIIα-Cre positive parent was the female to prevent transmission of Cre-recombinase through male germ cells.

We used male and female animals aged between 2.5 - 6 months exclusively for all experiments. The mice were housed in a vivarium that was maintained between 22 - 23°C with a 12-hr light on/off cycle. Food and water were accessible *ad libitum*. All experiments detailed here were reviewed and approved by the animal care committee of the University Health Network in accordance with the Canadian Guidelines for Animal Care.

### Method details

#### PCR for genotyping

Mouse tails were collected, and genomic DNA was obtained by alkaline lysing methods where the samples were lysed with 50 mM NaOH while incubating at 96°C for 1 hour with vigorous shaking. The supernatant was directly used in PCR for genotyping. PCR was performed to distinguish wild-type vs flox mice and presence of Cre recombinase was also tested. For 5’ PCR, a forward primer 5’- GGGGGTGAGTTGAAGTCATTGAAG - 3’ (primer 1) and a reverse primer 5’- AGCTTAAACGGGATGAACTCAGGC - 3’ (primer 2). Primers are located on the end of the end of exon 4 and near the start of intron 4, surrounding the closing loxP site. For Cre recombinase PCR, the forward primer of 5’- CCCCAAGCTCGTCAGTCAA - 3’ (primer 3) and the reverse primer of 5’- ACAGAAGCATTTTCCAGGTATGCT - 3’ (primer 4) were used.

#### Preparation of hippocampal sections for two photon imaging

Mice were anesthetized with an intraperitoneal injection of sodium pentobarbital (75 mg/kg, Bimeda-MTC) and transcardially perfused with 10 mL of PBS followed by 10 mL of 4% PFA. The brain was removed and postfixed overnight at 4°C in 4% PFA. The brain was then washed in PBS and dehydrated in 30% sucrose overnight. Brains were mounted in 50% OCT compound and 50% sucrose and 40 μm coronal sections were prepared using a cryostat. For immunohistology, slices were washed in PBS with 0.1% triton X-100. Rhodamine-phalloidin was incubated for 90 mins. After washing, slices were then blocked for one hour in PBS with 0.3% triton X-100 and 10% goat serum. Primary antibody was incubated overnight in blocking solution. Primary antibodies were used at 1:125 dilution (antibodies listed in key resources table). After washing, secondary antibody (A-11008) Alexa Fluor 488 Goat anti-Rabbit IgG or (A-11001) Alexa Fluor 488 Goat anti-Mouse IgG was incubated for 2 hours in blocking solution at 1:1000 dilution. Slices were then washed in PBS with 0.1% triton X-100, placed in PBS, and mounted in Mowiol.

#### Two-photon fluorescence imaging of CA1 hippocampus in hippocampal sections following anti-SNAP & phalloidin co-staining

Whole-brain coronal sections were prepared as described above. Fluorescence imaging of the CA1 hippocampus was conducted with a two-photon microscope (Custom made FV1000 MPE; Olympus, Tokyo, Japan, 60x objective lens, NA 1.0 equipped with Spectra-Physics InSight DeepSee; Spectra-Physics, CA, US). An 860 nm laser was used for excitation. Green (Alexa Fluor 488 for SNAP isoforms) and red (rhodamine phalloidin) fluorescence were imaged at 495-540 nm and 575-630 nm emission, respectively. The fluorescence image was combined across a z-stack composed of 6 slices, taken at 0.5 μm intervals for each slice.

#### Quantification of postsynaptic signal in two-photon fluorescence images

Image analysis was performed using Imaris (Bitplane, Belfast, UK). The fluorescence images were separated into green (SNAP isoforms) and red (phalloidin) fluorescence channels on Imaris. Regions of dendritic spines were manually selected on the images by following the accumulation of phalloidin (observed as red fluorescent puncta) along the primary apical dendrites. The postsynaptic phalloidin puncta within each region were detected by the Imaris spot function (diameter = 0.99 μm) and used as an index for dendritic spines.

For fluorescence intensity analysis of dendritic spines, the green (SNAP isoforms) and red (phalloidin) fluorescence intensities in each puncta were measured using the intensity mean of the spots function. The normalized accumulation of SNAP isoforms in dendritic spines was determined by the ratio of fluorescence intensity of green fluorescence (SNAP isoforms)/red fluorescence (phalloidin) in each puncta. Representative images were prepared from the maximum intensity projection of the z-stack images into a single plane image by Fluoview (Olympus, Tokyo, Japan).

#### Cresyl violet staining of hippocampal sections

Mice were anesthetized and prepared as described above, however transcardial perfusion was done with a 10% neutral buffered formalin solution (Sigma, Oakville, Canada). The brain was removed, postfixed, mounted, and a series of coronal cryosections were obtained at 40 μm thickness and stained with cresyl violet. Slices were imaged on a OMAX A3580U camera on a dissecting microscope with the OMAX ToupView v3.7 software. Quantification of hippocampus area and density was done in ImageJ, briefly, the hippocampus was manually selected and area and mean gray intensity were measured as a function of area and intensity respectively.

#### Preparation of hippocampal slices for electrophysiological recordings

Mice were anesthetized using an intra-peritoneal injection of sodium pentobarbital (75 mg/kg) and transcardially infused with ice-cold high sucrose dissection solution containing: 300 mM sucrose, 3.5 mM KCl, 2 mM NaH2PO4, 20 mM glucose, 0.5 mM CaCl2, 2 mM MgCl2 and 5 mM HEPES (pH adjusted to 7.4) before decapitation. The brain was quickly dissected and hemi-sectioned, and mid-line sides of two hemispheres of the brain were glued. Sagittal cortico-hippocampal slices of 400 μm thickness were obtained via a vibratome (VT1200, Leica Microsystems, Richmond Hill, Canada) in the presence of ice-cold high sucrose dissection solution and cortical tissues were removed leaving only the hippocampus. For each animal, an average of 10 - 12 hippocampal slices were sectioned. After sectioning, the slices were stabilized in artificial cerebrospinal fluid (ACSF) containing: 125 mM NaCl, 25 mM NaHCO3, 3.5 mM KCl, 1 mM NaH2PO4, 1 mM MgSO4, 2 mM CaCl2 with continuous gassing with 95% O2 and 5% CO2 at room temperature for at least 1 hour before the recording.

#### Electrophysiological recordings

The slice was placed in a submerged chamber and perfused with standard, oxygenated ACSF at a high flow rate of 15 mL/min. Both the top and bottom surfaces of the slice were exposed to ACSF. All recordings were done at room temperature. For all recordings, recording electrodes and stimulating electrodes were positioned under a dissecting microscope.

Recording electrodes were made with thin-wall glass tubes (World Precision Instruments, Sarasota, Florida). Extracellular electrodes were filled with a solution that contained 150 mM NaCl and 2 mM HEPES (pH 7.4; resistance of 1–2 MΩ). Electrodes for whole-cell current-clamp recordings were filled with solution that contained 140 mM potassium gluconate, 1 mM HEPES, 1 mM EGTA, 0.1 mM CaCl2, 0.5 mM NaGTP, 5 mM MgATP, and 5 mM phosphocreatine (pH 7.26 and resistance of ∼5 MΩ); electrodes for whole-cell voltage-clamp recordings were filled with solution that contained 120 mM cesium gluconate, 10 mM TEACl, 10 mM HEPES, 2 mM EGTA, 1 mM CaCl2, 1 mM MgCl2, 0.5 mM NaGTP, 5 mM MgATP, and 5 mM phosphocreatine (pH 7.18 and resistance ∼5 MΩ).

Extracellular and intracellular signals were recorded via a dual channel amplifier (700B, Molecular Devices/Axon Instruments, Sunnyvale, California). Data acquisition, storage and analysis were performed using PClamp software (version 10, Molecular Devices). These signals were recorded in frequencies of 0–5 kHz and digitized at 50 kHz (Digidata 1322A, Molecular Devices). For assessing evoked synaptic field potentials, extracellular recordings were made from the apical dendritic area of the CA1. For assessing intrinsic properties and spontaneous EPSC, CA1 somas were recorded via whole-cell patch clamp. Intrinsic properties were assessed via current clamp. Intracellular injections of squared current pulses of −300 pA to 500 pA in 25 pA steps and duration of 500 ms or intracellular injections of square current pulses of100 pA to 225 pA in 25 pA steps and duration of 50 ms were used to assess firing properties and spike properties. A bipolar electrode, made of polyimide-insulated stainless-steel wires (outer diameter 0.1 mm; Plastics One, Ranoake, Virginia), was placed in the *stratum radiatum* CA2 region for Schaffer collateral axon stimulation. Constant current pulses were generated by a Grass stimulator (S88H, Natus Neurology Incorporated – Grass Products, Warwick, Rhode Island) and delivered through an isolation unit.

For spontaneous EPSC recordings, the neurons were held at -70 mV via voltage clamp. Spontaneous EPSCS were analyzed using MiniAnalysis (Synaptosoft). For these voltage clamp recordings, we used a cesium-gluconate-based pipette solution which showed pipette resistance of 5 MΩ in standard ACSF. Only cells that showed stable responses for 10 minutes after establishing a whole-cell configuration were used.

#### Long-term potentiation induction

Stimulation at which results in 30% of the maximal evoked response of fEPSP slopes was quickly determined by giving constant current pulses (10–150 μA, 1 ms). Then the stability of evoked fEPSPs was monitored for 30 min prior to LTP induction. Slices which exhibited deviations of more than 10% in the baseline responses were rejected from further recordings. LTP was induced by applying 3 sec of continuous theta burst stimulation: 15 bursts of four pulses at 100 Hz, with an interburst interval of 200 msec. After theta burst stimulation, responses were further recorded for 60 min.

#### Pharmacological agents and blockade experiments

(2R)-amino-5-phosphonovaleric acid (AP5) and picrotoxin were obtained from Cayman Chemicals. CNQX was obtained from Tocris. APV was initially dissolved in ddH2O to a stock concentration of 10 mM; the final concentration applied was 50 μM. CNQX was dissolved in ddH2O to a stock concentration of 10 mM; the final concentration applied was 20 μM. Picrotoxin at 100 μM was added directly to the Mg2+-free ACSF and was dissolved with an extensive period of stirring. Briefly, baseline recordings were made under standard ACSF. Then, the bath solution was switched to Mg2+-free ACSF containing 100 μM picrotoxin while stimulating the Schaffer collateral axons every 30 sec to maximize glutamatergic activations and responses. Epileptiform responses with long lasting phase with multiple spikes started to appear after 15 min. AP5 was added to inhibit NMDAR-mediated responses, once the AMPAR only component of the response stabilized for > 5 sweeps, CNQX was added to inhibit all responses.

#### Morris water maze test

Mice received visible platform training for 3 days (4 trials per day) and hidden platform training for 12 days (4 trials per day). If the mice did not find the platform within 90 seconds, they were guided to the platform by the experimenter’s hand. For hidden platform training, the platform was submerged under 1.5 cm of water and the procedure is otherwise the same as visible platform training. The location of the platform was changed in hidden platform training compared to visible platform training. For reversal training, the location of the platform was changed to a different quadrant from the regular hidden platform training. On days 6, 9, 12 and 15, mice were given a 60 second probe trial where they were allowed to explore the maze without platform. The inter-trial interval was roughly 10–15 minutes. Learning was assessed by evaluating time and distance required to find the hidden platform in the training trials and memory was measured by examining time spent during the probe trials in the quadrant of the pool where the platform was previously located.

#### Marble burying test

Marble burying task was performed with minor modifications to a previously published protocol.^58^ Briefly, cages were half filled with 5 cm of corn bedding, and 20 marbles were lined approximately 2 cm apart within the cage. Mice were each placed on the bedding in a corner of the cage, and the cage lid closed. Mice were left undisturbed and allowed to freely dig around the cage for an hour, food and water were withheld during the test. At the end of the session, any marble at least two-thirds covered with bedding were scored as buried.

#### Nestlet shredding task

Nestlet shredding task was performed with minor modifications to a previously published protocol.^58^ Briefly, mice were placed in a cage with a single, preweighed nestlet, and the cage lid closed. Mice were left undisturbed and allowed to roam around the cage for an hour, food and water were withheld during the test. The mouse was returned to its home cage after test completion. Nestlet height (in cm) and nestlet volume (in cm3) were then measured.

#### Open-field test

Open field ambulation test was performed with minor modifications to a previously published protocol.^59^ Briefly, mice were placed in a plexiglass cage for an hour. An automated movement detection system recorded the motor activities of the animal. Measured parameters were distance traveled and rearing count.

### Quantification and statistical analysis

Statistics were performed using Origin Pro 2016 (OriginLab, Northampton, MA) and SPSS (IBM SPSS Statistics, Armonk, NY). All error bars represent SEM. For comparison of two groups, a two-sample t test was performed using Origin Pro. Mixed ANOVA testing was performed in SPSS. The scipy.stats package on Python was used for independent t-tests and Mann-Whitney U Rank tests. For all analyses, *p < 0.05* was considered to be statistically significant. Corresponding test details and n values are defined in figure captions. For all box plots, the median (central line), the 25th–75th percentiles (bounds of the box), and outliers (whiskers) are represented.

## Data Availability

• All data reported in this paper is available from the lead contact upon request. • This paper does not report original code. • Any additional information required to reanalyze the data reported in this paper is available from the lead contact upon request.
