# Supplementary material for: Neuronal SNAP-23 is critical for synaptic plasticity and spatial memory independently of NMDA receptor regulation
Source: iScience. 2023 Apr 13;26(5):106664. doi: 10.1016/j.isci.2023.106664 (PMC10165271; doi:10.1016/j.isci.2023.106664)
Supplement: Document S1. Figures S1–S4 [file mmc1.pdf]

iScience, Volume *26*

Supplemental information

Neuronal SNAP-23 is critical for synaptic plasticity and spatial memory independently of NMDA receptor regulation

Mengjia Huang, Na-Ryum Bin, Jayant Rai, Ke Ma, Chun Hin Chow, Sarah Eide, Hidekiyo Harada, Jianbing Xiao, Daorong Feng, Hong-Shuo Sun, Zhong-Ping Feng, Herbert Y. Gaisano, Jeffrey E. Pessin, Philippe P. Monnier, Kenichi Okamoto, Liang Zhang, and Shuzo Sugita

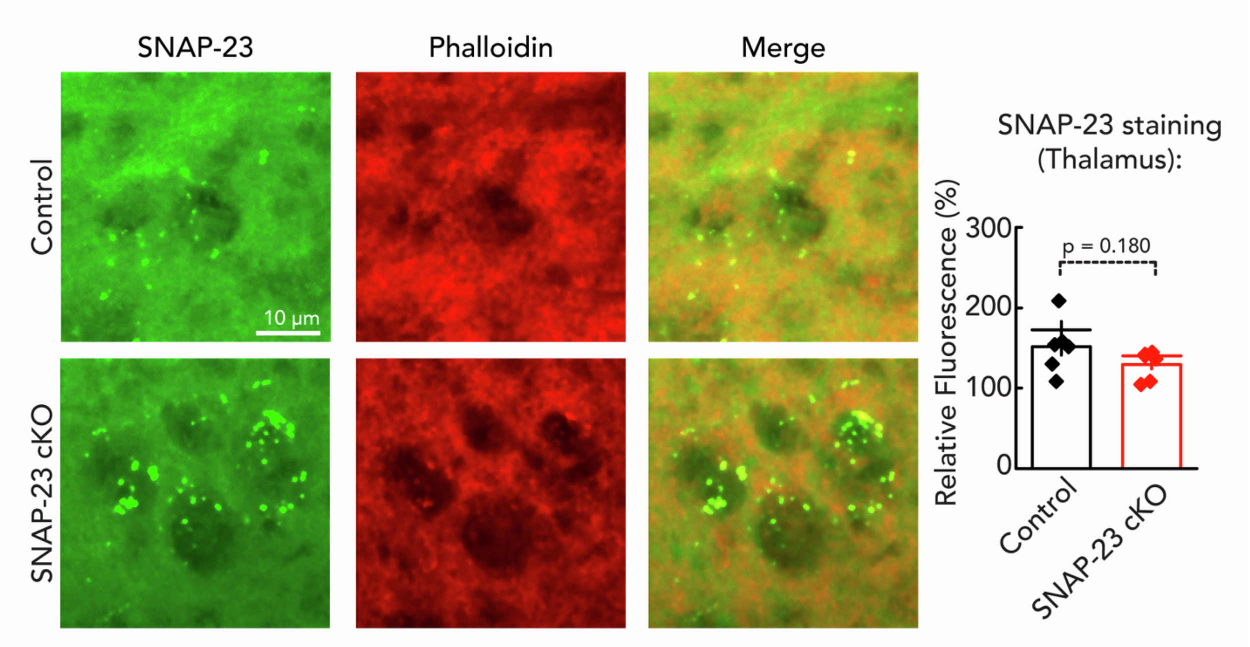

Supplemental Figure 1. SNAP-23 removal is specific to brain regions that express CaMKIIα-Cre, related to Figure 1.

SNAP-23 (green) costained with phalloidin (red) in the thalamic area. SNAP-23 staining is observed in the thalamic area but does not significantly decrease following SNAP-23 conditional removal (Mann-Whitney U rank test, $t_{(10)} = 27.0$, $p = 0.180$). Scale bar = 10 µm. Error bars represent SEM.

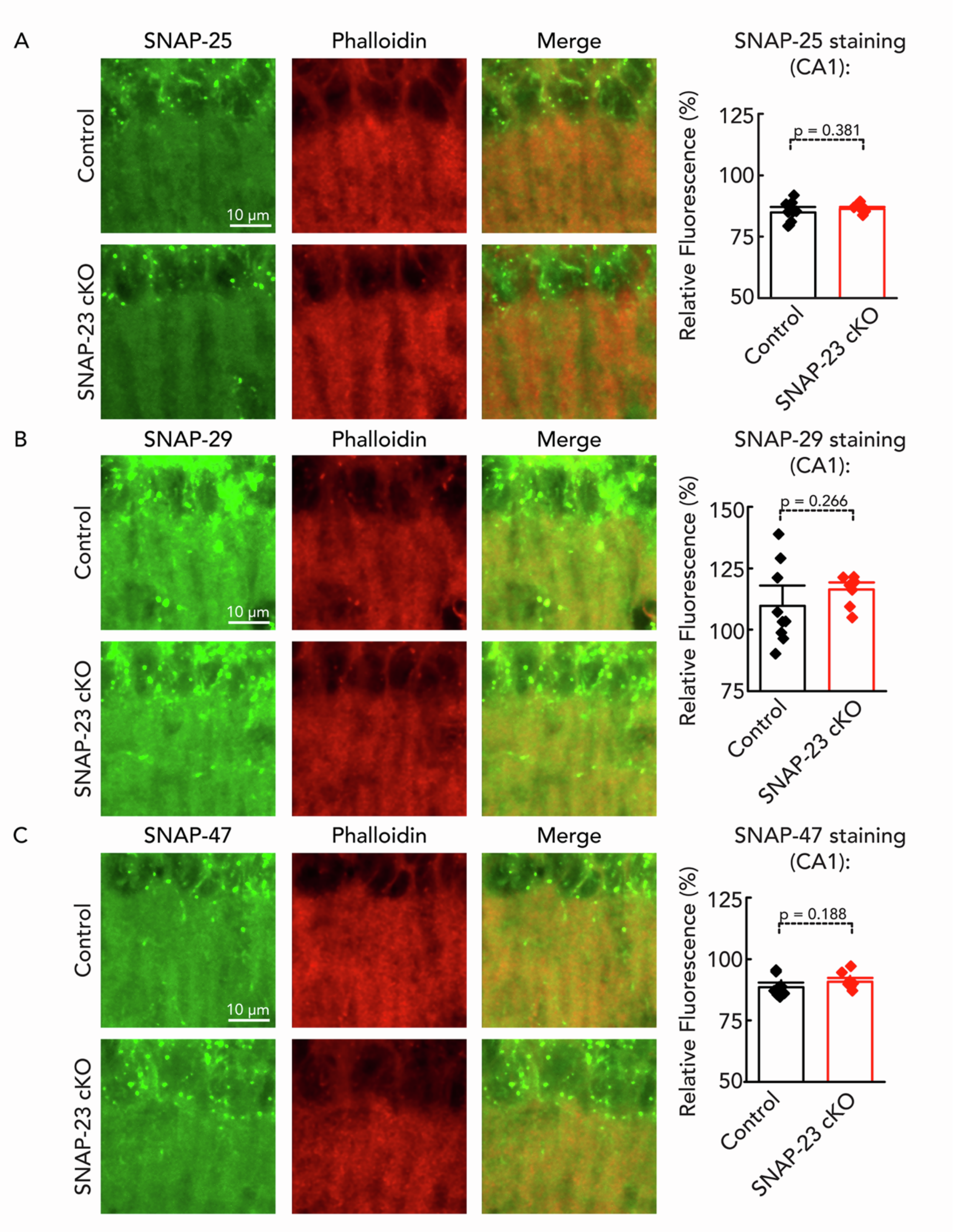

Supplemental Figure 2. Other SNAP family members are not upregulated to compensate for the absence of SNAP-23, related to Figure 1.

(A) SNAP-25 (green) and phalloidin (red) staining of control and SNAP-23 cKO CA1 dendrites. Relative fluorescence in percent is quantified on the right, no significant changes to SNAP-25 level is observed in the SNAP-23 cKO CA1 dendritic region compared to control (independent t-test, $t_{(16)} = -0.900$, $p = 0.381$). (B) SNAP-29 (green) and phalloidin (red) staining of control and SNAP-23 cKO CA1 dendrites. Relative fluorescence in percent is quantified on the right, no significant changes to SNAP-29 level is observed in the SNAP-23 cKO CA1 dendritic region compared to control ($t_{(16)} = -1.153$, $p = 0.266$). (C) SNAP-47 (green) and phalloidin (red) staining of control and SNAP-23 cKO CA1 dendrites. Relative fluorescence in percent is quantified on the right, no significant changes to SNAP-47 level is observed in the SNAP-23 cKO CA1 dendritic region compared to control ($t_{(16)} = -1.374$, $p = 0.188$). Scale bar = 10 μm for all images. Error bars represent SEM.

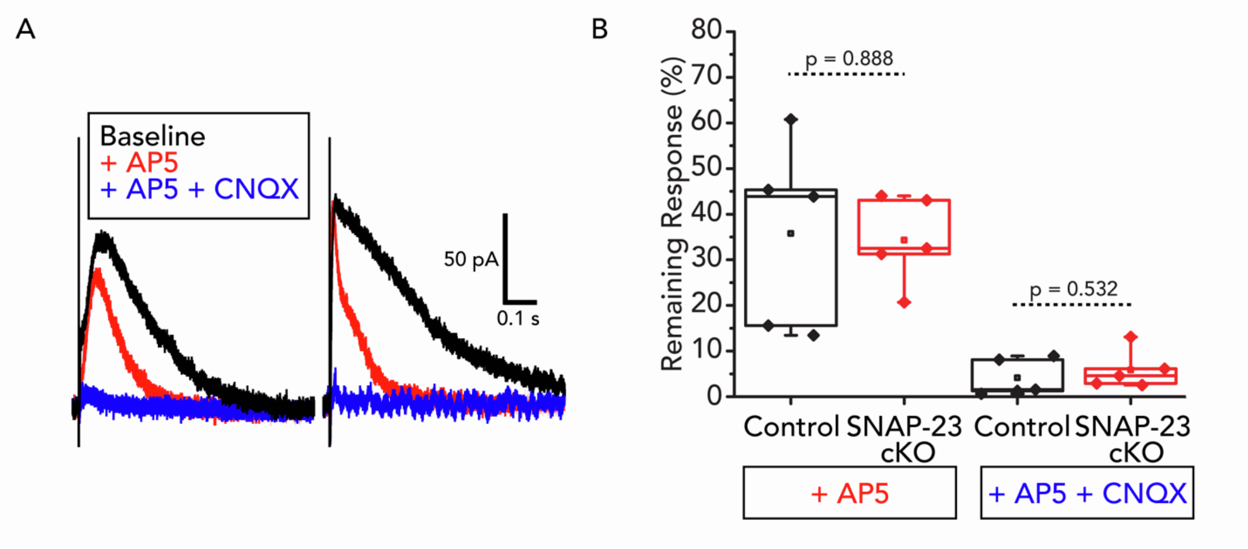

Supplemental Figure 3. Evoked response is decreased by AP5, and further eliminated by CNQX in both control and SNAP-23 cKO groups, related to Figure 3.

(A) Representative traces of voltage clamp experiments where CA1 pyramidal neurons were voltage clamped at +40 mV to observe both AMPAR and NMDAR-mediated responses. Recordings were done in 100 µM picrotoxin (PTX) containing ACSF. NMDAR antagonist AP5 was bath applied for 5 minutes to block the NMDAR component so only the AMPAR-mediated component remains (red). After stabilization of the AMPAR-mediated component, AMPAR antagonist CNQX was applied to remove remaining response (blue). (B) Normalized percent NMDAR and AMPAR charge transfer in control and SNAP-23 cKO mice. The area under the curve was calculated for both NMDAR and AMPAR components. The charge transfer was then normalized to the control group. No difference was observed in the SNAP-23 cKO mice compared to the control mice (independent t-test, after AP5: $t_{(8)} = 0.145$, $p = 0.888$, after AP5 + CNQX: $t_{(8)} = -0.654$, $p = 0.531$). Error bars indicate SEM.

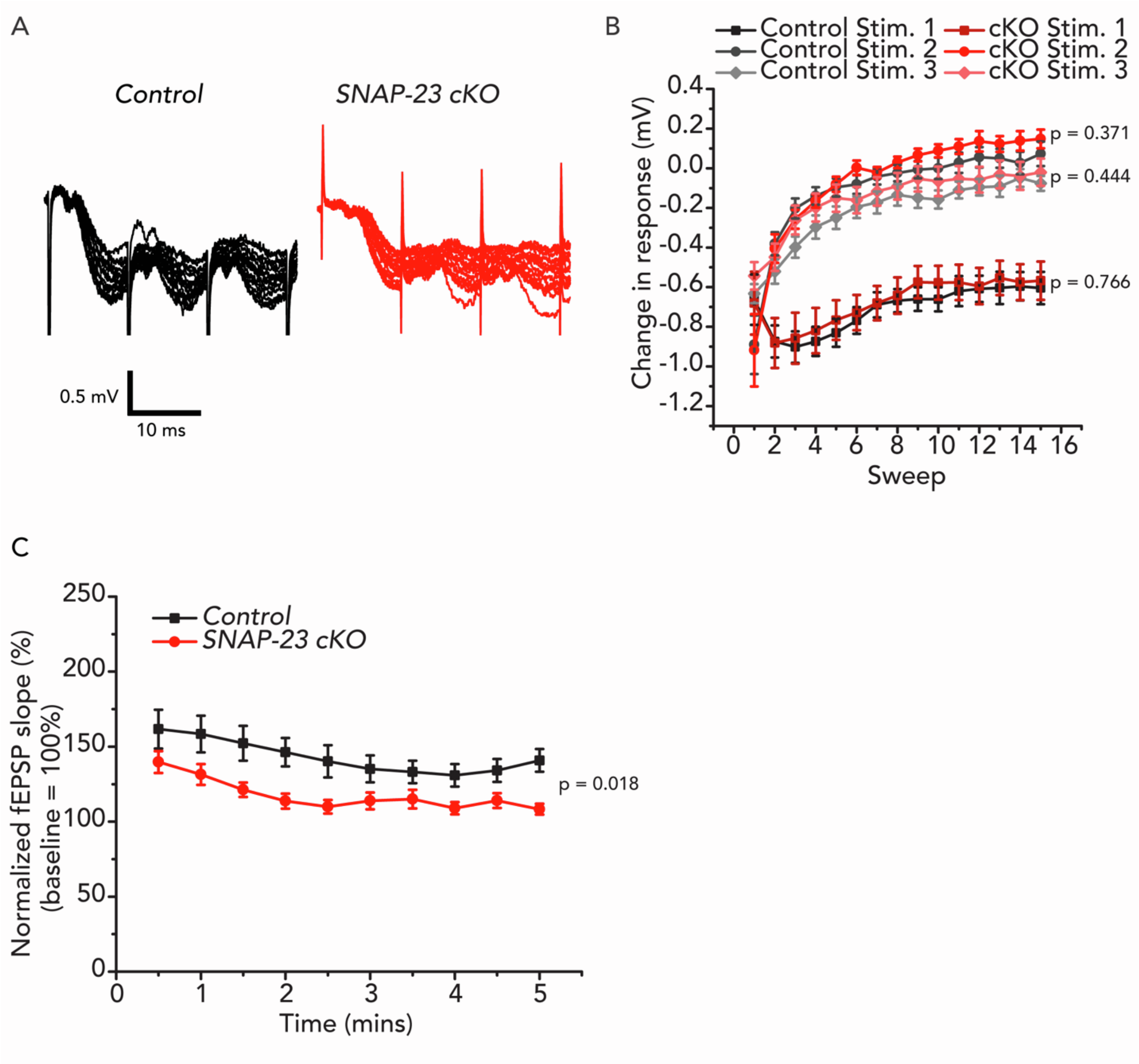

Supplemental Figure 4. Analysis of theta-burst induction and post-theta stimulation phase of LTP, related to Figure 6.

(A) Representative trace of extracellular slice response following theta burst stimulation (black trace control, red trace SNAP-23 cKO), 15 sweeps of four 100 Hz pulses were delivered to each slice to induce LTP. (B) Change in slice response (mV) during theta burst induction (greyscale control, reds SNAP-23 cKO). No difference was observed in slice response between both groups following first three stimulations (mixed ANOVA, stimulation 1 $F_{(1,21)} = 0.91$, $p = 0.766$,

stimulation 2 $F_{(1,21)} = 0.608$, $p = 0.444$, stimulation 3 $F_{(1,21)} = 0.835$, $p = 0.371$). Due to error in data acquisition, only first 3 stimulations were analyzed. (C) Normalized fEPSP slope (%) change during post tetanic phase of LTP maintenance (0.5 min – 5 mins after LTP induction). Group differences were observed for post-tetanic phase of LTP maintenance ($F_{(1, 15)} = 7.072$, $p = 0.018$). Error bars represent SEM.
